# Software evaluation on infant development to support teaching and professional training*

**DOI:** 10.1590/1518-8345.7248.4284

**Published:** 2024-08-19

**Authors:** Wesley Soares de Melo, Hévila Ferreira Gomes Medeiros Braga, Maria Vera Lúcia Moreira Leitão Cardoso, Emanuella Silva Joventino Melo, Flávia Paula Magalhães Monteiro

**Affiliations:** 1 Universidade Federal do Ceará, Fortaleza, CE, Brazil.; 2 Universidade da Integração Internacional da Lusofonia Afro-Brasileira, Redenção, CE, Brazil.

**Keywords:** Pediatric Nursing, Child development, Nursing education, Software, Educational Technology, Validation Study

## Abstract

**Objective:**

evaluate the functional performance and technical quality of the Wise Infant Development^®^ educational software with experts.

**Method:**

methodological research that followed the software evaluation process according to the ISO/IEC 25010 and NBR ISO-IEC 14598-6 standards. The software’s functional performance was assessed by a group of nurse experts and its technical quality by information technology experts. The Content Validity Index and the Binomial test were used for statistical analysis.

**Results:**

in both expert groups, agreement was greater than 70%, indicating that the software is suitable and pertinent to what was proposed in all its evaluated characteristics: functional suitability, reliability, usability, performance efficiency, compatibility, security, maintainability, and portability. The technology received suggestions for improvement, which were accepted.

**Conclusion:**

the Wise Infant Development^®^ software was well evaluated by the experts and could contribute to teaching about infant development, both in undergraduate nursing courses and in professional training.

## Highlights

**(1)** The software presents satisfactory technical quality and functional performance.

**(2)** Technology is useful in the students’ teaching-learning process.

**(3)** The software can be used to train child health professionals.

## Introduction

Technologies are becoming more and more integrated into our daily lives, which is why professionals have been seeking to develop and use innovative strategies ever since they were trained. In the midst of this, Digital Information and Communication Technologies (DICT) can be used as educational technologies, being useful as cognitive tools that contribute to the implementation of a hybrid or remote approach to teaching and learning^1^. 

During the social isolation experienced in the COVID-19 pandemic, the need to use technology has intensified, especially in education. It has become an essential tool to improve teaching and learning, as well as to show that knowledge can be accessible anywhere, at any time, adapting to the pace of each person^2^ ^)-(^ ^3^. 

In this sense, it can be seen that DICTs are also occupying space in nursing education, both at undergraduate and postgraduate level, as well as in professional training. The use of DICTs has shown numerous benefits in teaching and learning, such as the possibility of personalizing teaching, optimized processes, reduced drop-out rates, and technological innovations help develop clinical skills and support the decision-making process in health^4^ ^)-(^ ^5^.

In the context of children’s health, nurses need to rigorously assess children’s development, especially at childcare appointments, both for proper monitoring and for early interventions if any delays are identified. With this in mind, Wise Infant Development (WID^®^), an educational software program anchored to a web server, was developed using the JavaScript programming language and in the light of Piaget’s Genetic Epistemology Theory from a Cognitive Constructivist perspective^6^. 

This technology can help the teaching-learning process of future nurses, as well as professional training on infant development, with a view to improving child health care^7^. Nurses are responsible for caring and supporting children and their families, identifying and intervening in their needs and vulnerabilities^8^ ^)-(^ ^9^.

There are still professionals who do not adequately assess neuropsychomotor development or who confuse this assessment with the child’s general condition and growth measurements, failing to take into account actions that include assessing milestones and the risks of developmental delays for the child age. In this way, a proper understanding of child development assessment is a decisive criterion for qualified care and timely, targeted interventions^10^.

In this sense, training strategies should provide students and professionals with experiences that encourage them to reflect and act in the context of health care. Computer-mediated learning can add more interactivity to conventional educational approaches. Thus, the inclusion of software in the teaching and learning process can foster the acquisition of knowledge and overcome the shortcomings present in traditional approaches to assessing child development^7^.

However, any educational technology needs to go through a rigorous evaluation process before being applied to its intended audience. On this basis, the product’s evaluation process must ensure its technical, pedagogical and methodological support, in addition to guaranteeing satisfactory resources and functions, as well as sufficient and comprehensible content for the purpose for which it was developed^11^. 

Therefore, evaluating WID^®^’s quality becomes an important procedure to ensure that the software performs its functions properly and is approved according to credible technical standards.

Teaching using this technology can be a privileged moment for building knowledge in order to encourage the consolidation of a body of knowledge on the subject in question. Therefore, the aim of this study was to evaluate the functional performance and technical quality of Wise Infant Development-WID^®^, an educational software to support the teaching of infant development, with experts. 

## Method

### Study design

Methodological, technological evaluation study. The technology evaluated is the educational software called Wise Infant Development-WID^®^, which has been registered with the National Institute of Industrial Property (*Instituto Nacional de Propriedade Industrial*, INPI) and is therefore guaranteed to be valid in Brazil and in 176 other countries that are part of the Berne Convention (1886). The study was guided by the SQUIRE guidelines.

### Location and data collection period

The software was developed between January 2019 and February 2020, and the evaluation stage with the experts took place between February and March 2020, at a Higher Education Institution (HEI) located in the Redenção city, Ceará state, Brazil. 

### Participants and selection criteria

The selection of the expert panel followed the guidelines of NBR ISO/IEC 14598-6, a specific standard on the number of experts that recommends using at least eight members in each group of evaluators, in order to be representative of the category of software users^12^. The study therefore involved two groups of experts: 1. Nurses working in the areas of child health, child development and technologies; and 2. Professionals working in the field of information technology (IT) with an emphasis on software development/validation. 

To select the nurse experts, the following adapted criteria were considered: having a thesis and/or dissertation on the subject of Infant Child Development; having at least one year’s teaching experience in subjects in the area of Child Health; specializing in Pediatric Nursing and having completed a course on child development; having at least one year’s clinical practice in the area of Child Health; and having published research/articles on Child Health with content relevant to the area in question^13^. It is worth noting that the adaptation mentioned refers to the targeting of the criteria to the areas of interest in the study.

For the selection of the IT experts, the criteria established were: to have a thesis or dissertation on the subject of software engineering and/or systems analysis; to be specialized in software engineering area or a related area; to have scientific production on the subject of software engineering and/or systems analysis; to have developed software; to have professional experience of at least one year in systems analysis and/or software development^13^. 

The experts were identified from their CVs on the Lattes Platform by applying filters such as those relating to academic background, professional activity, mentoring activities, and presence in the directory of research groups for both groups of experts. Priority was given to professionals who demonstrated significant expertise in the specific areas mentioned^13^. 

In this process, the snowball technique was also used to identify potential experts. As soon as the authors contacted the professionals selected via the Lattes Platform by e-mail, they were invited to take part in the study and asked to recommend other experts who could help with the evaluation. 

All contact was made by e-mail. An invitation letter was sent to the experts’ e-mail addresses. A total of 70 professionals were invited, 19 of whom agreed to take part in the study. Once they had accepted, they were sent the Informed Consent Form (ICF) with the digital signature of the researcher responsible and asked to return it with the participant’s digital or scanned signature. The evaluators had 30 days to return their evaluations, during which time the software was available to the experts. Three participants were excluded due to loss of contact with the researchers. 

### Instruments used to collect information

The evaluation process was operationalized according to the international standard ISO/IEC 25010 (System and Software engineering-System and software Quality Requirements and Evaluation-SQuaRE-System and software quality models), which considers the characteristics of software product quality^14^ shown in Figure 1.

**Figure 1:** ISO/IEC 25010 characteristics of the software quality model

System/Software product quality		
Characteristics	Sub-characteristic	Definition
**Functional adequacy**	Functional integrity; Functional correctness; Functional fitness.	This relates to the need for the software’s functionalities to meet what was requested in its requirements.
**Reliability**	Maturity; Fault tolerance; Recoverability; Availability.	This refers to the software’s ability to maintain its performance level under established conditions over a period. This characteristic can be seen when the software, under certain conditions (e.g. resource scarcity), is able to perform its functions reliably.
**Usability**	Adequacy recognition; Apprehensibility; Protection against error; Operability; Aesthetics of the user interface; Accessibility.	It relates to the effort required to use the software, as well as the individual judgment of its use by a group of users. It indicates that the software can be used by specific users with certain levels of effectiveness, efficiency, and satisfaction.
**Performance efficiency**	Time; Resources; Capacity	Characteristic related to the software’s performance level and the amount of resources used, under established conditions.
**Compatibility**	Coexistence; Interoperability.	This relates to the quality of the product, system, or component, to exchange information with other products, systems, or components, and/or perform their necessary functions, while sharing the same hardware or software environment. The aim is for the software to be able to exchange information with other systems in the same operating environment.
**Security**	Confidentiality; Integrity; Non-repudiation; Accountability; Authentication.	It relates to the information and data protection and control of the access level of people, products, or systems according to the authorization types and levels. It is evident when the software protects its information and data according to established authorization levels.
**Maintainability**	Analyzability; Modifiability; Modularity; Reusability; Testability.	This refers to the effort required to make specified changes to the software.
**Portability**	Adaptability; Ability to navigate; Ability to substitute.	This relates to the software’s ability to be transferred from one environment to another. It checks that the software can be transferred to another operating environment defined in its requirements efficiently and effectively.

### Data collection

The software is organized into the following functions: basic functions, pre- and post-test functions, main software screen, information about the technology, teaching modules, tests, certificate, frequently asked questions, edit profile and software administration panel. 

As for the software’s basic functions, it offers essential features such as: the home screen for accessing the technology from the user’s ID and the options to: “Reset password”; and “Register new user”. After logging in, users are automatically directed to a welcome screen followed by information on a pre-test, consisting of 16 multiple-choice questions, used to assess students’ prior knowledge of child development. This strategy aims to measure the software’s effectiveness as an educational tool. After completing the pre-test, a screen informs them of the end of this stage and the total number of correct answers, without revealing which questions were answered correctly to avoid memory bias in the post-test.

After completing the pre-test, students are redirected to the main screen, which shows the students’ identification and offers the following navigation options: information about the technology, teaching modules, tests, certificate, frequently asked questions, edit profile and log out. The item on information about the technology provides a description of what the software is, its purpose and contribution to nursing practice.

The teaching modules are structured as follows: Module I - Introduction (4 lessons; 4 screens); Module II - Physical development (15 lessons; 42 screens); Module III - Cognitive development (6 lessons; 14 screens); Module IV - Psychosocial development (5 lessons; 5 screens); Module V - Child development in Brazil (6 lessons; 12 screens). The modules’ content is presented through texts and flowcharts that can be enlarged to optimize viewing and are complemented by photos and videos taken from the Brazilian Ministry of Health’s website. These can be accessed on the WID^®^ platform to further enhance the learning process. 

All the content is organized into numbered “Lessons”, providing a clear sense of where the content is and an effective distribution of it. This approach facilitates memorization and note-taking while using the software, with the intention of making it didactic in its presentation and format of use. 

It should be noted that the software’s content was created based on a literature review carried out by two reviewers in the following databases: Latin American and Caribbean Health Sciences Literature (LILACS), Medical Literature Analysis and Retrieval System Online (MEDLINE), Scientific Electronic Library Online (SciELO) and the Portal of the Coordination for the Improvement of Higher Education Personnel in Brazil (CAPES Portal - a platform that brings together national and international scientific productions). The controlled MeSH/DeCS terms “infant” and “infant development” and the uncontrolled terms “physical development”, “psychosocial development” and “cognitive development” were used in combination with the Boolean operator “AND”^7^.

Titles and abstracts were read and selected for full-text review. Scientific articles available electronically in full text in the chosen databases, published in Portuguese or English in the last 5 years, were included. Manuals, books, and official Ministry of Health publications were also considered. Duplicates and studies that did not address child development were excluded^7^.

The review included 22 articles, three manuals, seven books and one official publication from the Brazilian Ministry of Health^7^.

After completing all the modules, students are directed by the software to a post-test screen. It should be noted that the questions in the post-test are identical to those in the pre-test, to provide a comparative analysis and help assess the knowledge acquired. The layout of the questions in the post-test has been changed from the pre-test in order to reduce memorization.

In the “Tests” section, users can see the number of questions answered correctly in both the pre-test and post-test, once all the teaching modules have been completed. If this is not the case, the software notifies users that they must complete all the modules before they can start the post-test.

In relation to the certificate, the user has access to a completion certificate for the software-mediated course if they have completed all the modules and the post-test. The certificate attests to participation in the course and contains the student’s full name, the completion date, workload, details of the teaching modules, the software logo, and the educational institution responsible for the course. In addition, the Frequently Asked Questions section provides answers and appropriate support to help users with any questions they may have about the software.

The user profile contains the information provided during registration, and it is possible to edit these details at any time using the “Edit profile” function. There is also an administration panel designed for monitoring purposes. This allows the tutor or teacher to supervise progress and manage students’ activities in the software. The dashboard provides log data, including test performance, progress in the teaching modules and facilitates effective pedagogical mediation.

To collect data from the participants, we used an instrument that includes all the ISO/IEC 25010 characteristics, which was translated and adapted for evaluating software in research of this nature. The nurse experts evaluated six characteristics: functional suitability, reliability, usability, performance efficiency, compatibility, and security. The information technology experts, on the other hand, evaluated all eight of the characteristics indicated, plus the characteristics of maintainability and portability, as they have technical data specific to this group of experts. 

Each item evaluated was given a concept on a five-point ordinal scale with the following classifications: 1 - Not at all appropriate; 2 - Somewhat appropriate; 3 - Moderately appropriate; 4 - Very appropriate; 5 - Completely appropriate^15^. The researchers emailed the experts the following PDF files: interaction and navigation plans for the software with explanations of the technology’s architecture and instructions for use, as well as the evaluation instrument. Subsequently, the file was received in the same form online. 

The evaluation process of the features/sub-features analyzed was based on the NBR ISO-IEC 14598-6 standard^11^ adapted by Sperandio^16^, in which the author mentions the minimum value of 70% of indications as appropriate (very appropriate or completely appropriate) for the features/sub-features to be considered appropriate. 

### Data processing and analysis

The survey data was analyzed using the IBM Statistical Package for the Social Sciences (SPSS), version 26.0. The content validity index (CVI) and the binomial test were used with a proportion of 0.70, consisting of an expected agreement value equal to or greater than 70% with a significance level of 5% (alpha = 0.05)^17^.

### Ethical aspects

The study was approved by the Research Ethics Committee of the University of the International Integration of Afro-Brazilian Lusophony (*Universidade da Integração Internacional da Lusofonia Afro-Brasileira*, UNILAB), under opinion no. 3.465.662 and certificate of appreciation no. 08328319.5.0000.5576.

## Results

The sample consisted of 16 evaluators, eight in each group. In the nurses’ group, the majority of participants were female (n=7), of whom four had a doctorate, three a master’s degree and one a post-doctorate. The mean age was 35.6 (±6.36) years and the mean time working in the area was 13.1 (±6.79) years, with the predominant area of activity being teaching and research (n=6). This group was made up of experts from Brazil’s Northeast, Midwest, and South regions, working in different public and private HEIs. The group of IT professionals was predominantly male, with a mean age of 31.3 (± 4.20) years. Four had a master’s degree, three a doctorate and one a specialization. The mean time working in the area was 6.7 (±2.18) years, with the predominant areas of activity being teaching and teaching/research, with three experts in each one. All the experts were from Brazil’s Northeast region and worked in different public and private HEIs.

The results of both expert groups’ assessment of the ISO/IEC 25010 characteristics/sub-characteristics of the quality model indicate that the software is suitable and pertinent to what was proposed, with most questions showing 70% agreement. It can be seen that only the accessibility sub-characteristic did not reach adequate agreement (>70%) to be considered quality, requiring corrections prior to its application with the target audience, as shown in Table 1.

**Table 1:** Experts’ assessment based on ISO/IEC 25010 (n = 16). Redenção, CE, Brazil, 2020

Characteristics	Expert nurses			IT experts*		
	CVI^†^	Binomial	p-value	CVI^†^	Binomial	p-value
**Functional adequacy**						
Functional integrity	0.87	0.90	0.001	0.87	0.90	0.001
Functional correctness	0.87	0.90	0.001	0.87	0.90	0.001
Functional fitness	1.00	1.00	0.058	1.00	1.00	0.058
**Reliability**						
Maturity	0.75	0.80	0.011	0.75	0.80	0.011
Fault tolerance	1.00	1.00	0.058	0.75	0.80	0.011
Recoverability	1.00	1.00	0.058	0.87	0.90	0.011
Availability	1.00	1.00	0.058	0.87	0.90	0.011
**Usability**						
Adequacy recognition	1.00	1.00	0.058	0.87	0.90	0.001
Apprehensibility	1.00	1.00	0.058	1.00	1.00	0.058
Operability	0.87	0.90	0.001	0.87	0.90	0.001
Accessibility	0.00	0.00	0.058	0.12	0.10	0.255
Protection against error	1.00	1.00	0.058	0.87	0.90	0.001
Aesthetics of the user interface	1.00	1.00	0.058	1.00	1.00	0.058
**Performance efficiency**						
Time	1.00	1.00	0.058	0.87	0.90	0.001
Resources	1.00	1.00	0.058	0.87	0.90	0.001
Capacity	1.00	1.00	0.058	1.00	1.00	0.058
**Compatibility**						
Interoperability	0.87	0.90	0.001	1.00	1.00	0.058
Coexistence	1.00	1.00	0.058	0.87	0.90	0.001
**Security**						
Confidentiality	1.00	1.00	0.058	0.75	0.80	0.011
Integrity	1.00	1.00	0.058	0.87	0.90	0.011
Non-repudiation	0.87	0.90	0.001	0.87	0.90	0.011
Accountability	1.00	1.00	0.058	1.00	1.00	0.058
Authentication	0.87	0.90	0.001	0.87	0.90	0.001
**Maintainability**						
Analyzability	-	-	-	0.75	0.80	0.011
Modifiability	-	-	-	1.00	1.00	0.058
Modularity	-	-	-	0.87	0.90	0.001
Testability	-	-	-	0.75	0.80	0.011
Reusability	-	-	-	1.00	1.00	0.058
**Portability**						
Adaptability	-	-	-	1.00	1.00	0.058
Ability to navigate	-	-	-	1.00	1.00	0.058
Ability to substitute	-	-	-	1.00	1.00	0.058

*IT = Information technology; ^†^CVI = Content validity index

Only five characteristics received suggestions from the experts about the functional performance and technical quality of the WID^®^, which were accepted in the process of improving the technology evaluated, as shown in Figure 2. 

**Figure 2:** Experts’ suggestions on evaluating the functional performance and technical quality of the WID^®^

Nurse experts	IT* experts
**Functional adequacy**	
The physical development content covers skin folds in infants. Some renowned authors consider it important to take this measurement. In cognitive development, specify the types of language the infant has (receptive and non-receptive) and what is expected at each stage.	Perhaps it would be interesting to include videos and activities to show the student’s progress. Because, as it is only content, the student can go through all the pages without reading what is in the content.
Make the text more interactive for the reader. Ask questions and stimulate reflection, not just in the exercises, but throughout the text.	The administrative panel could have a search for users and pagination. As the number of users grows, the dashboard table can become difficult to maintain.
Present drawings to emphasize how the assessments should be carried out, in addition to the videos. Show in the drawings the important repair points that the picture doesn’t cover, for example, how to measure the head circumference.	There should be more mechanisms for assessing whether students have actually read the content and whether they have managed to assimilate what they have been taught.
Make available which questions in the post-test had the wrong answer, as well as the correct answer sheet, in order to encourage learning.	To repair the progress of the modules on the start screen, which was sometimes not displayed and prevented the post-test from being carried out.
	To repair navigation problems between modules. When the software stopped, it was necessary to refresh the page and then connect again.
**Reliability**	
Notice that every time you complete a module, the software takes you to the post-test.	To repair glitches in the “next lesson” button between teaching modules II and III.
Improve speed, it is slow to load the next lessons. After connecting again, it worked better.	Correct errors when taking the post-test. The message “student not found” appeared.
Repair minor glitches: the software crashed and when I refreshed the page, I had to log in again. But when I logged in again, I didn’t have to redo the pre-test.	Repair failures during the modules’ execution so that you don’t have to go back to the initial screen to continue the learning process.
	The software sometimes fails to save the activities’ progress. Make a technical correction.
Repair errors in the execution of the post-test.	When you lose connection during a lesson and click on next lesson, the button disappears and keeps loading endlessly. Ideally, the next lesson button should return and show the user a message.
**Usability**	
Greater interaction in each module, with clinical cases, photos, videos, and reflection exercises.	At the end of each module, set activities to assess the content learned.
Reduce writing and make more playful schemes.	Have resources such as Anno, aSimpleTour, Bootstrap.js, among others, that explain the main sections of the system.
	The administrative panel could have greater control over users (delete user, change password, update data, block user, etc.).
	Provide features such as increasing/decreasing the font, changing the contrast, access keys, etc.
Increase font and spacing. Put more pictures as examples.	Provide “magnifiers” that operate only in the software and a “speaker” so that the text can be read.
	Improve the layout’s responsiveness.
	Report the error that filling in the field is mandatory.
**Performance efficiency**	
Enlarge video. It’s too small in the top right corner of the screen.	To increase the use of video and audio resources, essential elements in a teaching platform.
**Security**	
Identify the access date, as well as the author, in the admin panel.	The HTTPS^†^ protocol is not used. An essential protocol for using logins and passwords. I suggest changing it.
	To suggest that the user enters a secure password, stating the minimum number of characters, letters, at least one capital letter, numbers, and special characters.
	Improve the login request. It is sent in the body of the request and is accessible by any malicious software.
	Identify records with date and time of access.

*IT = Information technology; ^†^HTTPS = Hyper Text Transfer Protocol Secure

## Discussion

All educational software needs to undergo an evaluation prior to its implementation in the educational context. This evaluation aims to identify whether the technology in question has satisfactory characteristics in terms of pedagogical aspects, involving the quality of the proposed content, the interface in terms of usability, and elements related to technical and functional quality^11^. In this context, it was observed that WID^®^ achieved satisfactory results in its functional performance and technical quality in the experts’ evaluation.

A study on the analysis of quality requirements for hard technologies in the field of health education identified functional adequacy as one of the main points affecting software quality^18^. In relation to this characteristic, the experts asked for greater content implementation and for it to be approached with a more attractive and dynamic content, using drawings, images, including more activities throughout the teaching modules, as well as general repairs to the technology.

Considering that infant development involves a series of progressive and complex transformations, influenced by internal and external factors and the care environment, it is crucial to understand and highlight in teaching technologies the unique characteristics of infant development in order to promote their health in achieving their global abilities. As a result, it is essential to employ technologies that can help health professionals and students to monitor children’s development^19^ ^)-(^ ^20^. In addition, educational resources that incorporate audiovisual elements are able to captivate the viewer in a multisensory way, offering interactivity and promoting a more effective understanding of the content, enriching the learning experience^21^. 

Reliability analysis is essential in order to act preventively in the event of possible failures and guarantee greater availability of the technology for use^22^ ^)-(^ ^23^. In this respect, the experts, especially those in the information technology’s area, contributed important suggestions for repairs to be made to the WID^®^ in order to improve it. This implies that reliability analysis is a fundamental practice to ensure that systems and equipment remain operational and reliable, minimizing interruptions and impacts on the market. 

In this technology, technical repairs will be needed to run the lessons in the teaching modules, improve the navigation speed and content loading, possible errors related to user registration and improve the storage and progress of activities already carried out by students.

The WID^®^ usability proved to be acceptable based on the experts’ evaluation. This means that the software is pleasant and meets the needs for which it is intended. Usability tests are becoming increasingly fundamental in the evaluation process, as they seek to improve the user experience^24^ ^)-(^ ^25^. This is why the expert participation is so important, especially those in the information technology area, as they have mastered this characteristic and have therefore carried out a thorough inspection and testing in order to guarantee a good product, with the aim of ensuring the quality and acceptance of WID^®^.

The other considerations given to usability in general, such as: the need for greater interaction in each module with clinical cases, photos, videos, playful schemes and reflection exercises; at the end of each module, activities to evaluate the content learned, as well as the technical repairs mentioned, go hand in hand with the suggestions given to functional suitability, as both are related to the extent to which the software fulfills what is proposed, also guaranteeing a good user experience. 

The resources mentioned, such as Anno, aSimpleTour, Bootstrap.js, were suggested with the intention of providing functions to explain the main sections of the system and how to navigate the software. Another point that needs attention is the responsiveness of the WID^®^ layout, which is related to the technology’s ability to adapt to different types of environments and screens, such as smartphones and tablets.

Although the WID^®^ was well evaluated in terms of usability, the accessibility sub-characteristic of the WID^®^ for people with visual impairments was considered inappropriate and received important suggestions from both expert groups, including: providing resources such as increasing/decreasing the font, changing the contrast, access keys, increasing the font and spacing, providing “magnifying glasses” that operate only in the software and a “sound box” so that the content can be audio-described. However, it should be noted that this technology was designed to be used by people with low visual acuity (partial visual impairment). It is possible to use the browser’s own zoom feature, which will automatically increase the font size of the letters and images in the software, making it easier to read and navigate.

The performance efficiency characteristic was rated satisfactorily by the experts. The agility criterion is recognized as highly important and unique when evaluating software performance based on the efficiency and/or effectiveness of the actions proposed in the technology^26^ ^)-(^ ^27^. A study carried out on evaluating and comparing software highlights that performance efficiency is a crucial point when choosing software and implementing it in the environment for which it was proposed^28^.

The evaluation by the experts was fundamental, as in addition to quantifying agreement, difficulties in the technology’s performance were identified and improvement actions were suggested to improve WID^®^, all of which were accepted and resolved in the software’s final version, such as: the ability to enlarge videos and implement media resources, as these are essential elements in a teaching platform. This improvement is necessary because the learning process has become more dynamic and flexible with the advent of technologies that provide students with different methodologies, resources and methods^29^.

Depending on the complexity of the technology developed, there are several challenges to be overcome if compatibility is to be fully achieved in healthcare systems, and it is essential that professionals in the areas of technology/software development and healthcare work together to advance this feature^30^. On this matter, the suggestions made by the experts contribute to improving minor flaws between the WID^®^ modules and the networked system, which, although they have not had a major impact on technology, also have repercussions on compatibility.

WID^®^ received important suggestions from experts to improve the software’s security, which were duly accepted. The quality of the security feature acts on the integrity of the information, in other words, on preventing attacks on the data to ensure that the systems are re-established and that secure access to the information is guaranteed even when attacks on the computer system are successful^31^. WID^®^ received important suggestions from experts to improve the software’s security, which were duly accepted. The quality of the security feature acts on the integrity of the information, in other words, on preventing attacks on the data to ensure that the systems are re-established and that secure access to the information is guaranteed even when attacks on the computer system are successful. In this sense, it is important to adopt measures to minimize malicious intrusions when adopting the HTTPS (Hypertext Transfer Protocol Secure) access protocol, such as: identifying the date users access the administrative panel; reinforcing security for the use of logins and passwords and ensuring that the password contains upper- and lower-case letters, numbers, and special characters.

In view of this, the software development based on security standards is essential to reduce information loss and save effort and operating costs^32^. Therefore, WID^®^ was concerned with security from the initial software lifecycle stages so that this requirement was well thought out, taking into account data authentication, the database and the backup system.

Maintainability is related to the ease with which software can be modified throughout its development, from product corrections to requirements adaptations. For this reason, it is desirable to adopt good practices throughout software development that benefit its maintainability^33^. Good coding practices were therefore used to develop WID^®^, with a view to its adherence, use and ease of maintenance in educational institutions. As a result, the software’s maintainability was rated highly by the technical experts. 

A relevant factor in the success of a software development is the technology’s ability to adapt to the changes that occur with a certain frequency in the health field as a result of advances in knowledge, which has a positive impact on the maintainability and suitability of the technology^34^. In this way, the lower the effort/cost in the software maintenance cycle, the higher its quality tends to be^35^.

As for portability, WID^®^ was developed with a system architecture with applications in JavaScript (a high-level programming language interpreter of what you want to put in the software and the potential for migration), which establishes a programming link with the server and database. In addition, the software is hosted on an Amazon Web Services (AWS) cloud server. 

These characteristics are relevant since a technology is only considered portable if it can be run on different platforms, involving modifying or maintaining the software so that it adapts and runs in a new environment. This is the process of moving software from one platform to another. When the application is developed using the Java programming language, there are better quality indicators due to its portability and dynamism^35^.

The WID^®^ software’s contributions to the advancement of scientific knowledge in nursing are related to the provision of a reliable and valid extension and support tool to favor the teaching-learning process of students and health professionals on infant development.

A limitation is that the technology was not developed specifically for people with disabilities, and this feature could be included in future software updates. In addition, in relation to the methodological aspect, data was only collected online; however, it is believed that the option of face-to-face collection could have been given to experts who live in the same region as the researchers.

## Conclusion

WID^®^ was validated using the software quality metrics indicated by ISO/IEC 25010 and NBR ISO-IEC 14598-6 and was considered adequate in terms of functional performance and technical quality. It should be noted that the improvement of this technology does not end with the suggestions and evaluation of the experts. This is an ongoing process that is part of a software’s life cycle. Therefore, new ideas and updates may emerge with the implementation and daily use of this tool in nursing education.

It is suggested that further studies be carried out on the WID^®^ based on the technology’s intervention effects in order to validate it with the target audience of undergraduate nursing students. In addition, studies could be carried out to apply this software in training and development programs for professionals, especially those who work in primary health care.
