# Supplementary material for: Software evaluation on infant development to support teaching and professional training*
Source: Rev Lat Am Enfermagem. 2024 Aug 19;32:e4284. doi: 10.1590/1518-8345.7248.4284 (PMC11335069; doi:10.1590/1518-8345.7248.4284)
Supplement: Translation

# Translation: Avaliação de *software* sobre o desenvolvimento do lactente para apoio ao ensino e capacitações profissionais*

**DOI:** 10.1590/1518-8345.7248.4285

**Authors:** Wesley Soares de Melo, Hévila Ferreira Gomes Medeiros Braga, Maria Vera Lúcia Moreira Leitão Cardoso, Emanuella Silva Joventino Melo, Flávia Paula Magalhães Monteiro

**Affiliations:** 1 Universidade Federal do Ceará, Fortaleza, CE, Brasil.; 2 Universidade da Integração Internacional da Lusofonia Afro-Brasileira, Redenção, CE, Brasil.

**Keywords:** Enfermagem Pediátrica, Desenvolvimento Infantil, Educação em Enfermagem, Software, Tecnologia Educacional, Estudo de Validação

## Abstract

**Objetivo:**

avaliar junto a *experts* o desempenho funcional e a qualidade técnica do *software* educativo *Wise Infant Development* ^*®*^ .

**Método:**

pesquisa metodológica que seguiu o processo de avaliação de *software*s conforme as normas ISO/IEC 25010 e NBR ISO-IEC 14598-6. O desempenho funcional do *software* foi avaliado por grupo de *experts* enfermeiros e a qualidade técnica por *experts* da tecnologia da informação. Para a análise estatística, empregou-se o Índice de Validade de Conteúdo e o teste Binomial.

**Resultados:**

em ambos os grupos de *experts*, a concordância foi maior que 70%, indicando que o *software* é adequado e pertinente ao que foi proposto em todas as suas características avaliadas: adequação funcional, confiabilidade, usabilidade, eficiência de desempenho, compatibilidade, segurança, manutenibilidade e portabilidade. A tecnologia recebeu sugestões para seu aperfeiçoamento, as quais foram aceitas.

**Conclusão:**

o *software Wise Infant Development* ^*®*^ foi bem avaliado pelos *experts*, podendo contribuir para o ensino sobre desenvolvimento do lactente, tanto na graduação em enfermagem quanto nas capacitações profissionais.

## Destaques

**(1)** O *software* apresenta qualidade técnica e desempenho funcional satisfatórios.

**(2)** A tecnologia é útil no processo de ensino-aprendizagem dos alunos.

**(3)** O *software* pode ser utilizado para capacitar profissionais na área de saúde infantil.

## Introdução

As tecnologias estão cada vez mais integradas em nosso cotidiano, de forma que os profissionais vêm, desde a sua formação, buscando desenvolver e utilizar estratégias inovadoras. Em meio a isso, as Tecnologias Digitais de Informação e Comunicação (TDIC) podem ser utilizadas como tecnologias educacionais, sendo úteis como ferramentas cognitivas que contribuem para a implantação de uma abordagem híbrida ou remota de ensino e de aprendizagem^1^. 

Durante o isolamento social vivido na pandemia da COVID-19, a necessidade do uso de tecnologias se intensificou, sobretudo no meio educacional. Tornou-se uma ferramenta essencial para aprimorar o ensino e aprendizagem dos indivíduos, bem como para mostrar que o conhecimento pode ser acessível em qualquer lugar, a qualquer momento, adequando-se ao ritmo de cada pessoa^2^ ^)-(^ ^3^. 

Nesse sentido, observa-se que as TDIC vêm também ocupando espaços no ensino em enfermagem, tanto na graduação quanto na pós-graduação, bem como em capacitações profissionais. O uso das TDIC tem demonstrando inúmeros benefícios no ensino-aprendizagem, tais como possibilidade de personalizar o ensino, processos otimizados, redução da evasão, além disso, as inovações tecnológicas colaboram no desenvolvimento de habilidades clínicas e apoiam o processo de tomada de decisão em saúde^4^ ^)-(^ ^5^.

No contexto da saúde da criança, os enfermeiros precisam avaliar rigorosamente o desenvolvimento infantil, sobretudo nas consultas de puericultura, tanto para um acompanhamento adequado quanto para intervenções precoces, caso algum atraso seja identificado. Diante disso, foi desenvolvido o *Wise Infant Development* (WID^®^), um *software* educacional, ancorado a um servidor *web*, desenvolvido sob a linguagem de programação *JavaScript* e à luz da Teoria da Epistemologia Genética de Piaget, na perspectiva Cognitiva Construtivista^6^. 

A referida tecnologia pode auxiliar o processo de ensino-aprendizagem de futuros enfermeiros, bem como as capacitações profissionais sobre desenvolvimento infantil do lactente, com vistas a melhorar assistência à saúde da criança^7^. O enfermeiro tem responsabilidades de cuidado e apoio à criança e seus familiares, identificando e intervindo em necessidades e vulnerabilidades^8^ ^)-(^ ^9^.

Ainda há profissionais que não avaliam o desenvolvimento neuropsicomotor adequadamente ou que confundem essa avaliação com o estado geral da criança e as mensurações de crescimento, não contemplando as ações que englobam a avaliação dos marcos e riscos para atraso no desenvolvimento, segundo a idade apresentada. Desse modo, a adequada compreensão da avaliação do desenvolvimento infantil é um critério decisivo para uma assistência qualificada e com intervenções oportunas e direcionadas^10^.

Nesse sentido, estratégias para capacitações devem proporcionar aos estudantes e profissionais experiências que os instiguem a refletir e a agir no contexto do cuidado em saúde. A aprendizagem mediada por computador pode adicionar mais interatividade às abordagens convencionais no âmbito da educação. Assim, a inclusão de um *software* no processo de ensino e aprendizagem pode propiciar a aquisição de conhecimento e superar as carências presentes nas abordagens tradicionais de avaliação do desenvolvimento infantil^7^.

Todavia, qualquer tecnologia educativa precisa passar por um rigoroso processo de avaliação, antes de ser aplicada ao público para o qual se destina. Com base nisso, é preciso que o processo de avaliação do produto assegure seu aporte técnico, pedagógico e metodológico, além de garantir os recursos e funções satisfatórios, bem como conteúdos suficientes e compreensíveis para o objetivo ao qual foi desenvolvida^11^. 

Assim, avaliar a qualidade do WID^®^ se torna um procedimento importante para garantir que o *software* execute suas funções adequadamente e seja aprovado conforme normas técnicas de credibilidade.

O ensino proporcionado por meio desta tecnologia poderá ser um momento privilegiado de construção do saber a fim de estimular a consolidação de um corpo de conhecimento sobre a temática em questão. Nesse sentido, o presente estudo teve como objetivo avaliar junto a *experts* o desempenho funcional e a qualidade técnica do *Wise Infant Development-*WID^®^, *software* educativo para o apoio no ensino do desenvolvimento do lactente. 

## Método

### Delineamento do estudo

Estudo metodológico, de avaliação tecnológica. A tecnologia avaliada é o *software* educacional denominado *Wise Infant Development-*WID^®^, o qual possui registro junto ao Instituto Nacional de Propriedade Industrial-INPI e, perante isso, há a garantia de sua validade no Brasil e em outros 176 países que fazem parte da Convenção de Berna (1886). O estudo foi norteado pelas diretrizes SQUIRE.

### Local e período da coleta de dados

O *software* foi desenvolvido no período de janeiro de 2019 a fevereiro de 2020, e a etapa de avaliação com os *experts* ocorreu nos meses de fevereiro a março de 2020, em uma Instituição de Ensino Superior (IES), localizada no município de Redenção, Ceará, Brasil. 

### Participantes e critérios de seleção

A seleção do quadro de *experts* obedeceu às orientações da NBR ISO/IEC 14598-6, uma norma específica sobre o quantitativo de *experts* que preconiza a utilização de pelo menos oito membros em cada grupo de avaliadores, para haver representatividade da categoria de usuários do *software* ^12^. Desse modo, o estudo contou com a participação de dois grupos de *experts*: 1. Enfermeiros atuantes nas áreas de saúde da criança, desenvolvimento infantil e tecnologias; e 2. Profissionais atuantes na área de tecnologia da informação (TI) com ênfase no desenvolvimento/validação de *software*. 

Para a seleção dos *experts* enfermeiros, consideraram-se os critérios adaptados: ter tese e/ou dissertação direcionada para a temática de Desenvolvimento Infantil do Lactente; ter experiência docente de pelo menos um ano em disciplinas da área de Saúde da Criança; ter especialização em Enfermagem Pediátrica com trabalho de conclusão de curso voltado para desenvolvimento infantil; possuir prática clínica de pelo menos um ano de duração, na área da Saúde da Criança; ter publicação de pesquisa/artigos sobre Saúde da Criança com conteúdo relevante para a área em foco^13^. Vale ressaltar que a adaptação mencionada se refere ao direcionamento dos critérios para as áreas de interesse do estudo.

Já para seleção dos *experts* da TI, os critérios adaptados estabelecidos foram: possuir tese ou dissertação com a temática de engenharia de *software* e/ou análise de sistemas; ter especialização na área de engenharia de *software* ou área afim; possuir produção científica com a temática de engenharia de *software* e/ou análise de sistemas; ter desenvolvimento de *softwares*; possuir experiência profissional de pelo menos um ano em análise de sistemas e/ou desenvolvimento de *softwares* ^13^. 

Os *experts* foram identificados a partir dos currículos da Plataforma Lattes por meio da aplicação de filtros, tais como relacionados à formação acadêmica, atuação profissional, atividades de orientação e presença no diretório de grupos de pesquisas para ambos os grupos de *experts*. Foram priorizados profissionais que demonstraram *expertise* significativa nas áreas específicas mencionadas^13^. 

Nesse processo, também foi empregada a técnica de *snowball* para identificar potenciais *experts*. A partir do momento que os autores entraram em contato via *e-mail* com os profissionais selecionados através da Plataforma Lattes, estes eram convidados a participarem do estudo e solicitadas indicações de outros *experts* que colaborassem na avaliação. 

Todo o contato na pesquisa foi realizado por correio eletrônico. Foi enviada uma carta-convite para o *e-mail* dos *experts*. Ao todo, foram convidados 70 profissionais, dos quais 19 aceitaram participar do estudo. Após o aceite, foi enviado o Termo de Consentimento Livre e Esclarecido (TCLE) contendo a assinatura digital do pesquisador responsável, sendo solicitada a devolução com a assinatura digital ou escaneada do participante. Os avaliadores tiveram um prazo de 30 dias para a devolutiva de suas avaliações, período no qual o *software* estava com acesso liberado para os *experts*. Foram excluídos três participantes por perda de contato com os pesquisadores. 

### Instrumentos utilizados para a coleta das informações

O processo de avaliação foi operacionalizado conforme a norma internacional ISO/IEC 25010 (*System and Software engineering-System and software Quality Requirements and Evaluation-SQuaRE-System and software quality models*), que considera as características da qualidade do produto de *software* ^14^ apresentadas na Figura 1.

**Figura 1:** Características da ISO/IEC 25010 do modelo de qualidade do *software*

**Sistema/Qualidade de Produto de *Software***		
Característica	Subcaracterística	Definição
**Adequação funcional**	Integridade funcional; Correção funcional; Aptidão funcional.	Relaciona-se à necessidade das funcionalidades do *software* atender ao que foi solicitado em seus requisitos.
**Confiabilidade**	Maturidade; Tolerância a falhas; Recuperabilidade; Disponibilidade.	Relaciona-se à capacidade de o *software* manter seu nível de desempenho sob condições estabelecidas durante um período. Percebe-se tal característica quando o *software*, sob determinadas condições (exemplo: escassez de recursos), consegue executar suas funcionalidades de forma confiável.
**Usabilidade**	Reconhecimento de adequação; Apreensibilidade; Proteção contra erro; Operabilidade; Estética da interface do usuário; Acessibilidade.	Relaciona-se ao esforço necessário para utilizar o *software*, bem como ao julgamento individual de seu uso, por um conjunto de usuários. Indica que o *software* pode ser utilizado por usuários específicos, com níveis determinados de eficácia, eficiência e satisfação.
**Eficiência de desempenho**	Tempo; Recursos; Capacidade	Característica relacionada entre o nível de desempenho do *software* e a quantidade de recursos usados, sob condições estabelecidas.
**Compatibilidade**	Coexistência; Interoperabilidade.	Refere-se à qualidade do produto, sistema ou componente para trocar informações com outros produtos, sistemas ou componentes, e/ou executar suas funções necessárias, enquanto compartilham o mesmo ambiente de *hardware* ou *software.* Almeja-se que o *software* possa trocar informações com outros sistemas no mesmo ambiente operacional.
**Segurança**	Confidencialidade; Integridade; Não repúdio; Responsabilização; Autenticação.	Relaciona-se à proteção de informações e dados e ao controle do nível de acesso de pessoas, produtos ou sistemas conforme os tipos e níveis de autorização. Evidencia-se quando o *software* protege suas informações e dados de acordo com níveis de autorização estabelecidos.
**Manutenibilidade**	Analisabilidade; Modificabilidade; Modularidade; Reusabilidade; Testabilidade.	Relaciona-se ao esforço necessário para fazer modificações especificadas do *software*.
**Portabilidade**	Adaptabilidade; Capacidade de navegação; Capacidade para substituir.	Relaciona-se à capacidade de o *software* ser transferido de um ambiente para outro. Verifica se o *software* pode ser transferido para outro ambiente operacional definido nos seus requisitos com eficiência e eficácia.

### Coleta de dados

O *software* está organizado nas seguintes funções: funções básicas, funções pré e pós-teste, tela principal do *software*, informações sobre a tecnologia, módulos de ensino, testes, certificado, perguntas frequentes, editar perfil e painel de administração do *software.*

Quanto às funções básicas do *software*, ele oferece funções essenciais, tais como: a tela inicial para acesso à tecnologia a partir da identificação do usuário e as opções de: “Redefinir senha”; e “Registrar novo usuário”. Após o *login*, os usuários são direcionados automaticamente para uma tela de boas-vindas seguidas de informações sobre um pré-teste, composto por 16 perguntas de múltipla escolha, utilizado para avaliar o conhecimento prévio dos alunos sobre o desenvolvimento infantil. Esta estratégia visa medir a eficácia do *software* como ferramenta educacional. Após a conclusão do pré-teste, uma tela informa o término desta etapa e o número total de respostas corretas, sem revelar quais perguntas foram respondidas corretamente para evitar viés de memória no pós-teste.

Após a conclusão do pré-teste, os alunos são redirecionados para a tela principal, que apresenta a identificação dos alunos e oferece as seguintes opções de navegação: informações sobre a tecnologia, módulos de ensino, testes, certificado, perguntas frequentes, editar perfil e sair. O item sobre as informações sobre a tecnologia fornece uma descrição do que é o *software*, seu propósito e contribuição para a prática de enfermagem.

No que se refere aos módulos de ensino, eles foram estruturados da seguinte maneira: Módulo I - Introdução (quatro aulas; quatro telas); Módulo II - Desenvolvimento físico (15 aulas; 42 telas); Módulo III - Desenvolvimento cognitivo (seis aulas; 14 telas); Módulo IV - Desenvolvimento psicossocial (cinco aulas; cinco telas); Módulo V - Desenvolvimento infantil no Brasil (seis aulas; 12 telas). O conteúdo dos módulos é apresentado por meio de textos e fluxogramas que podem ser ampliados para otimizar a visualização, sendo complementados por fotos e vídeos extraídos do site do Ministério da Saúde do Brasil. Esses podem ser acessados na plataforma WID^®^ para aprimorar ainda mais o processo de aprendizado. 

Todo o conteúdo é organizado em “Lições” numeradas, proporcionando uma sensação clara de localização do conteúdo e uma distribuição eficaz dele. Tal abordagem facilita a memorização e a tomada de notas durante a utilização do *software*, com a intenção de torná-lo didático em sua apresentação e formato de uso. 

Ressalta-se que o conteúdo do *software* foi criado a partir de uma revisão de literatura realizada por dois revisores nas seguintes bases de dados: Literatura Latino-Americana e do Caribe em Ciências da Saúde (LILACS), *Medical Literature Analysis and Retrievel System Online* (MEDLINE), *Scientific Electronic Library Online* (SciELO) e Portal da Coordenação de Aperfeiçoamento de Pessoal de Nível Superior no Brasil (Portal CAPES - plataforma que reúne produções científicas nacionais e internacionais). Os termos MeSH/DeCS controlados “*infant*” e “*infant development*” e os termos não controlados “*physical development*”, “*psychosocial development*” e “*cognitive development*” foram usados combinados pelo operador booleano “AND”^7^.

Os títulos e resumos foram lidos e selecionados para revisão do texto completo. Foram incluídos artigos científicos disponíveis eletronicamente em texto completo, nas bases de dados escolhidas, publicados em português ou inglês, nos últimos cinco anos. Também foram considerados manuais, livros e publicações oficiais do Ministério da Saúde. Foram excluídos duplicatas e estudos que não abordavam o desenvolvimento infantil^7^.

Foram incluídos na revisão 22 artigos, três manuais, sete livros e uma publicação oficial do Ministério da Saúde do Brasil^7^.

Após a conclusão de todos os módulos, os alunos são direcionados pelo *software* para uma tela de pós-teste. Destaca-se que as perguntas apresentadas no pós-teste são idênticas às do pré-teste, visando proporcionar uma análise comparativa e contribuir para a avaliação do conhecimento adquirido. A disposição das perguntas no pós-teste é alterada em relação ao pré-teste, a fim de mitigar a memorização.

Na seção denominada “Testes”, os usuários têm a possibilidade de consultar o número de perguntas respondidas corretamente tanto no pré-teste quanto no pós-teste, uma vez que todos os módulos de ensino tenham sido integralmente percorridos. Em situações contrárias, o *software* notifica os usuários de que é imperativo concluir todos os módulos para que o pós-teste possa ser iniciado.

Sobre o certificado, o usuário tem acesso ao certificado de conclusão do curso mediado pelo *software*, caso este tenha concluído todos os módulos e o pós-teste. O certificado atesta a participação no curso, nele contém o nome completo do aluno, a data de conclusão, a carga horária, detalhes sobre os módulos de ensino, o logotipo do *software* e a instituição de ensino responsável pelo curso. Além disso, na seção de Perguntas Frequentes exibe respostas e suporte apoio adequado para auxiliar os usuários em caso de dúvidas sobre o *software*.

O perfil do usuário contém as informações fornecidas durante o registro, sendo possível editar esses detalhes a qualquer momento por meio da função “Editar perfil”. Existe, adicionalmente, um painel de administração designado para fins de monitoramento. Esse permite ao tutor ou professor supervisionar o progresso e gerenciar as atividades dos alunos no *software*. O painel fornece dados de registro, incluindo desempenho nos testes, progresso nos módulos de ensino e facilita uma mediação pedagógica eficaz.

Para a coleta de dados com os participantes foi utilizado um instrumento que contempla todas as características da ISO/IEC 25010, o qual foi traduzido e adaptado para a avaliação de *softwares* em pesquisas dessa natureza, sendo que os *experts* enfermeiros avaliaram seis características: adequação funcional, confiabilidade, usabilidade, eficiência de desempenho, compatibilidade e segurança. Já os *experts* da área da tecnologia da informação avaliaram todas as oito características indicadas, ou seja, acrescidas das características manutenibilidade e portabilidade por possuírem dados técnicos específicos desse grupo de *experts*. 

Foi atribuído a cada item avaliado um conceito disposto em escala ordinal de cinco pontos com as seguintes classificações: 1 - Nem um pouco apropriado; 2 - Um pouco apropriado; 3 - Moderadamente apropriado; 4 - Muito apropriado; 5 - Completamente apropriado^15^. Os pesquisadores enviaram via *e-mail* aos *experts* os seguintes arquivos em PDF: planos de interação e de navegação do *software* com explicações sobre a arquitetura da tecnologia e as instruções de uso, bem como o instrumento de avaliação. Posteriormente, o arquivo foi recebido preenchido da mesma forma *online*. 

O julgamento do processo de avaliação das características/subcaracterísticas analisadas foi conduzido com base na norma NBR ISO-IEC 14598-6^11^ adaptada por Sperandio^16^, no qual o autor menciona o valor mínimo de 70% de indicação como apropriadas (muito apropriado ou completamente apropriado) para as características/subcaracterísticas serem consideradas adequadas. 

### Tratamento e análise dos dados

Os dados da pesquisa foram analisados utilizando o programa IBM *Statistical Package for the Social Sciences* (SPSS), versão 26.0. Empregou-se o índice de validade de conteúdo (IVC) e o teste binomial com proporção de 0,70, consistindo em um valor de concordância esperado igual ou superior a 70% com nível de significância de 5% (alfa = 0,05)^17^.

### Aspectos éticos

O estudo foi aprovado pelo Comitê de Ética em Pesquisa da Universidade da Integração Internacional da Lusofonia Afro-Brasileira (UNILAB), sob parecer de n.º 3.465.662 e certificado de apreciação n.º 08328319.5.0000.5576.

## Resultados

A amostra foi composta por 16 avaliadores, oito em cada grupo. No grupo de enfermeiros, a maioria dos participantes era do sexo feminino (n=7), destas: quatro possuem doutorado, três mestrado e uma pós-doutorado. Possui média de idade de 35,6 (±6,36) anos e a média de tempo de atuação na área foi de 13,1 (±6,79) anos, sendo a área de atuação predominante a de ensino e pesquisa (n=6). Esse grupo foi composto por *experts* das regiões Nordeste, Centro-Oeste e Sul do Brasil, atuantes em diferentes IES públicas e privadas. Já no grupo dos profissionais de TI, predominou o sexo masculino, com idade média de 31,3 (± 4,20) anos. Quatro possuem mestrado, três doutorado e um com especialização. A média do tempo de atuação na área foi de 6,7 (±2,18) anos, sendo as áreas de atuação predominantes a de ensino (37,5%) e ensino/pesquisa (37,5%), com três *experts* em cada. Todos os *experts* foram da região Nordeste do Brasil, atuantes em diferentes IES públicas e privadas.

Os resultados da avaliação de ambos os grupos de *experts* acerca das características/subcaracterísticas da ISO/IEC 25010 do modelo de qualidade indicam que o *software* é adequado e pertinente ao que foi proposto, com a maioria das questões apresentando 70% de concordância. Observa-se que apenas a subcaracterística acessibilidade não atingiu a concordância adequada (>70%) para ser considerada de qualidade, necessitando de correções prévias à sua aplicação com o público-alvo, conforme a Tabela 1.

**Tabela 1:** Avaliação dos *experts* com base na ISO/IEC 25010 (n = 16). Redenção, CE, Brasil, 2020

Características	***Experts* enfermeiros**			*Experts* da TI*		
	IVC^†^	Binomial	*p-valor*	IVC^†^	Binomial	*p-valor*
**Adequação funcional**						
Integridade funcional	0,87	0,90	0,001	0,87	0,90	0,001
Correção funcional	0,87	0,90	0,001	0,87	0,90	0,001
Aptidão funcional	1,00	1,00	0,058	1,00	1,00	0,058
**Confiabilidade**						
Maturidade	0,75	0,80	0,011	0,75	0,80	0,011
Tolerância a falhas	1,00	1,00	0,058	0,75	0,80	0,011
Recuperabilidade	1,00	1,00	0,058	0,87	0,90	0,001
Disponibilidade	1,00	1,00	0,058	0,87	0,90	0,001
**Usabilidade**						
Reconhecimento de adequação	1,00	1,00	0,058	0,87	0,90	0,001
Apreensibilidade	1,00	1,00	0,058	1,00	1,00	0,058
Operabilidade	0,87	0,90	0,001	0,87	0,90	0,001
Acessibilidade	0,00	0,00	0,058	0,12	0,10	0,255
Proteção contra erro	1,00	1,00	0,058	0,87	0,90	0,001
Estética de interface do usuário	1,00	1,00	0,058	1,00	1,00	0,058
**Eficiência de desempenho**						
Tempo	1,00	1,00	0,058	0,87	0,90	0,001
Recursos	1,00	1,00	0,058	0,87	0,90	0,001
Capacidade	1,00	1,00	0,058	1,00	1,00	0,058
**Compatibilidade**						
Interoperabilidade	0,87	0,90	0,001	1,00	1,00	0,058
Coexistência	1,00	1,00	0,058	0,87	0,90	0,001
**Segurança**						
Confidencialidade	1,00	1,00	0,058	0,75	0,80	0,011
Integridade	1,00	1,00	0,058	0,87	0,90	0,001
Não repúdio	0,87	0,90	0,001	0,87	0,90	0,001
Responsabilização	1,00	1,00	0,058	1,00	1,00	0,058
Autenticação	0,87	0,90	0,001	0,87	0,90	0,001
**Manutenibilidade**						
Analisabilidade	-	-	-	0,75	0,80	0,011
Modificabilidade	-	-	-	1,00	1,00	0,058
Modularidade	-	-	-	0,87	0,90	0,001
Testabilidade	-	-	-	0,75	0,80	0,011
Reusabilidade	-	-	-	1,00	1,00	0,058
**Portabilidade**						
Adaptabilidade	-	-	-	1,00	1,00	0,058
Capacidade para navegação	-	-	-	1,00	1,00	0,058
Capacidade para substituir	-	-	-	1,00	1,00	0,058

*TI = Tecnologia da informação; ^†^IVC = Índice de validade de conteúdo

Apenas cinco características receberam sugestões dos *experts* acerca do desempenho funcional e qualidade técnica do WID^®^, as quais foram aceitas no processo de melhoria da tecnologia avaliada, conforme a Figura 2. 

**Figura 2:** Sugestões dos *experts* acerca da avaliação do desempenho funcional e qualidade técnica do WID^®^

***Experts* enfermeiros**	*Experts* da TI*
**Adequação funcional**	
No conteúdo do desenvolvimento físico abordar as pregas cutâneas do lactente. Alguns autores renomados consideram importante realizar essa medida. No desenvolvimento cognitivo especificar os tipos de linguagem que o lactente possui (receptiva e não receptiva) e o que é esperado em cada etapa.	Talvez fosse interessante incluir vídeos e atividades para mostrar o progresso do discente. Pois, como somente conteúdo, o discente pode passar por todas as páginas sem ler o que tem nos conteúdos.
Proporcionar texto mais interativo para o leitor. Que faça perguntas e estimule a reflexão, não somente nos exercícios de fixação, mas, ao longo do texto.	O painel administrativo poderia ter uma busca por usuários e paginação. Enquanto o número de usuários cresce, a tabela do painel pode ficar difícil de manter.
Apresentação de desenhos para enfatizar como as avaliações devem ser realizadas, além dos vídeos. Mostrar nos desenhos os pontos importantes de reparo que a figura não aborda, por exemplo, como medir o perímetro cefálico.	Deveria ter mais mecanismos para avaliar se o discente leu realmente o conteúdo e se ele conseguiu assimilar o que lhe foi ensinado.
Disponibilizar quais questões do pós-teste tiveram a resposta errada, bem como o gabarito correto, a fim de favorecer o aprendizado.	Reparar o progresso dos módulos na tela inicial, que por vezes não era exibido, e impedia a execução do pós-teste.
	Reparar problemas de navegação entre os módulos. Quando o *software* parava, era necessário atualizar a página e, com isso, conectar novamente.
**Confiabilidade**	
Reparar pequeno erro que toda vez que completamos um módulo, o *software* já direciona para o pós-teste.	Reparar falhas no botão “próxima aula” entre os módulos de ensino II e III.
Melhorar velocidade, lentidão para carregar próximas aulas. Depois que conectei novamente, deu certo numa velocidade melhor.	Corrigir erros quanto à realização do pós-teste. Aparecia a mensagem “estudante não encontrado”.
Reparar falhas pequenas: o *software* travou e, quando atualizei a página, tive que fazer o login novamente. Mas quando conectei novamente, não tive que refazer o pré-teste.	Reparar falhas durante a execução dos módulos para não ser necessário voltar para a tela inicial para continuar o processo de aprendizagem.
	O *software* apresenta momentos em que não consegue salvar o progresso das atividades. Realizar correção técnica.
Reparar erros na execução do pós-teste.	Ao perder uma conexão durante uma aula e clicar em próxima aula, o botão some e fica carregando infinitamente. O ideal seria mostrar uma mensagem ao usuário e voltar o botão de próxima aula.
**Usabilidade**	
Maior interação a cada módulo, com casos clínicos, fotos, vídeos, exercícios de reflexão.	Ao fim de cada módulo, colocar atividades de avaliação do conteúdo aprendido.
Reduzir a escrita e fazer mais esquemas lúdicos.	Ter recursos como *Anno*, *aSimpleTour*, *Bootstrap.js*, entre outros, que explicassem as principais seções do sistema.
	O painel administrativo poderia ter maior controle sobre os usuários (excluir usuário, alterar senha, atualizar dados, bloquear usuário, etc.).
	Disponibilizar recursos como aumentar/diminuir fonte, alterar contraste, teclas de acesso, entre outros.
Aumentar letra e espaçamento. Colocar mais figuras como exemplo.	Disponibilizar “lupas” que operem apenas no *software* e “caixa de som” para poder haver uma leitura do texto.
	Melhorar a responsividade no *layout*.
	Informar o erro de que o preenchimento do campo é obrigatório.
**Eficiência de desempenho**	
Ampliar vídeo. Fica muito pequeno no canto superior direito da tela.	Aumentar o uso dos recursos de vídeos e áudio, elementos essenciais em uma plataforma de ensino.
**Segurança**	
Identificar data do acesso, além do autor, no painel administrativo.	Não é usado o protocolo HTTPS^†^. Protocolo essencial para o uso de *logins* e senhas. Sugiro alterar.
	Sugerir que o usuário digite uma senha segura, informando o número mínimo de caracteres, letras, pelo menos uma maiúscula, números e caracteres especiais.
	Melhorar a requisição de *login*. Esta é enviada no corpo da requisição, sendo acessível por qualquer *software* malicioso.
	Identificar registros com data e hora dos acessos.

*TI = Tecnologia da informação; ^†^HTTPS = *Hyper Text Transfer Protocol Secure*

## Discussão

Todo *software* educativo precisa ser submetido a uma avaliação prévia à sua implantação no contexto educacional. Esta avaliação visa identificar se a tecnologia em questão possui características satisfatórias quanto aos aspectos pedagógicos, envolvendo a qualidade do conteúdo proposto, a interface no eixo da usabilidade, os elementos relacionados à qualidade técnica e funcional propriamente ditas^11^. Nesse contexto, observou-se que o WID^®^ alcançou resultados satisfatórios em seu desempenho funcional e qualidade técnica na avaliação dos *experts*. 

Estudo realizado sobre análise dos requisitos de qualidade em tecnologias duras no âmbito do ensino em saúde identificou a adequação funcional como um dos principais pontos que afetam a qualidade dos *softwares* ^18^. No que se refere a essa característica, os *experts* solicitaram maiores implementações voltadas para o conteúdo e que este fosse abordado com um teor mais atrativo e dinâmico, por meio de desenhos, imagens, incluir mais atividades ao longo dos módulos de ensino, além de reparos gerais na tecnologia.

Considerando que o desenvolvimento infantil envolve uma série de transformações progressivas e complexas, influenciadas por fatores internos, externos e o ambiente de cuidado, é crucial compreender e destacar nas tecnologias de ensino as características únicas do desenvolvimento de lactentes para promover sua saúde no alcance de suas habilidades globais. Como resultado, torna-se essencial empregar tecnologias que possam ajudar profissionais e estudantes na área da saúde a supervisionar o desenvolvimento das crianças^19^ ^)-(^ ^20^. Outro assim, recursos educacionais que incorporam elementos audiovisuais conseguem cativar o espectador de maneira multissensorial, oferecendo interatividade e promovendo uma compreensão mais eficaz do conteúdo, enriquecendo a experiência de aprendizagem^21^. 

A análise de confiabilidade é fundamental para que se possa atuar preventivamente nas possíveis falhas e garantir maior disponibilidade da tecnologia para o uso^22^ ^)-(^ ^23^. Neste quesito, os *experts*, em especial os da área de tecnologia da informação, contribuíram com importantes sugestões de reparos a serem feitos no WID^®^ para seu aperfeiçoamento. Isso implica que a análise da confiabilidade é uma prática fundamental para garantir que os sistemas e equipamentos se mantenham operacionais e confiáveis, minimizando interrupções e impactos no mercado. 

Na presente tecnologia, serão necessários reparos técnicos de execução das aulas dos módulos de ensino, melhorar a velocidade de navegação e carregamento dos conteúdos, possíveis erros relacionados ao cadastro dos usuários e, aperfeiçoar o armazenamento e progresso das atividades já realizadas pelos alunos.

A usabilidade do WID^®^ se mostrou aceitável com base na avaliação dos especialistas. Esse fato permite afirmar que o *software* é agradável e atende às necessidades para o qual se destina. Os testes de usabilidade tornam-se cada vez mais fundamentais no processo de avaliação, pois desse modo buscam melhorar a experiência do usuário^24^ ^)-(^ ^25^. Por isso, a relevância da participação dos *experts*, em especial os da área de tecnologia da informação, ao dominarem essa característica e, por isso, realizaram uma inspeção apurada e testes a fim de garantir um bom produto, visando assegurar a qualidade e a aceitação do WID^®^.

As demais considerações dispensadas para a usabilidade de modo geral, tais como: a necessidade de uma maior interação a cada módulo com casos clínicos, fotos, vídeos, esquemas lúdicos e exercícios de reflexão; ao final de cada módulo colocar atividades de avaliação do conteúdo aprendido, além de reparos técnicos mencionados, vão ao encontro das sugestões dadas a adequação funcional, pois, ambas se relacionam à medida que o *software* cumpre ao que é proposto, garantindo também uma boa experiência ao usuário. 

Os recursos mencionados, como o *Anno*, *aSimpleTour*, *Bootstrap.js*, foram sugeridos na intenção de fornecer funções que buscassem explicar as principais seções do sistema e como navegar no *software*. Além disso, outro ponto que carece de atenção é a responsividade no *layout* do WID^®^, que está relacionada à capacidade da tecnologia se adaptar a diferentes tipos de ambientes e telas, como, por exemplo, em *smartphones* e *tablets.*

Apesar de o WID^®^ ter sido bem avaliado na usabilidade, a subcaracterística acessibilidade do WID^®^ para pessoas com deficiência visual foi tida como imprópria e recebeu importantes sugestões de ambos os grupos de *experts*, dentre elas: disponibilizar recursos como aumentar/diminuir fonte, alterar contraste, teclas de acesso, aumentar letra e espaçamento, disponibilizar “lupas” que operem apenas no *software* e “caixa de som” para poder haver audiodescrição do conteúdo. Porém, ressalta-se que a presente tecnologia foi planejada para ser usada por pessoas com baixa acuidade visual (deficiente visual parcial), sendo possível utilizar o recurso de zoom do próprio navegador que, automaticamente, aumentará o tamanho da fonte das letras e imagens dispostas no *software*, facilitando a legibilidade e navegação.

A característica eficiência de desempenho obteve avaliação satisfatória pelos *experts*. O critério agilidade é reconhecido com alta importância e singularidade ao avaliar o desempenho de *software* com base na eficiência e/ou na eficácia das ações propostas na tecnologia^26^ ^)-(^ ^27^. Estudo realizado sobre avaliação e comparação entre *softwares* destaca que a eficiência de desempenho é um ponto crucial para a escolha do *software* e implementação no meio para o qual foi proposto^28^.

A avaliação pelos *experts* foi fundamental, pois além da quantificação da concordância, foram identificadas dificuldades de desempenho da tecnologia e sugeridas ações de melhoria para aperfeiçoar o WID^®^, tendo sido todas aceitas e sanadas na versão final do *software*, tais como: a capacidade de ampliar vídeos e de implementar recursos de mídia, pois estes são elementos essenciais em uma plataforma de ensino. Esse aperfeiçoamento é necessário, visto que o processo de aprendizagem tem se tornado mais dinâmico e flexível com o advento das tecnologias que proporcionam aos educandos diferentes metodologias, recursos e métodos^29^.

Conforme a complexidade da tecnologia desenvolvida, são vários os desafios a serem superados para a compatibilidade poder ser totalmente alcançada nos sistemas em meio ao campo da saúde, sendo essenciais profissionais das áreas de tecnologia/desenvolvimento de *softwares* e de saúde trabalharem em conjunto para o avanço dessa característica^30^. Neste quesito, as sugestões atribuídas pelos *experts* contribuem para as melhorias de pequenas falhas entre os módulos do WID^®^ e do sistema em rede, que apesar de não terem causado grandes impactos pela tecnologia, repercutem também na compatibilidade.

O WID^®^ recebeu sugestões importantes dos *experts* para aperfeiçoar a segurança do *software*, as quais foram devidamente aceitas. A qualidade da característica segurança atua sobre a integridade das informações, ou seja, sobre a prevenção de ataques aos dados para assegurar que os sistemas sejam restabelecidos e que o acesso seguro às informações seja garantido mesmo quando houver êxito em ataques ao sistema computacional^31^. Nesse sentido, faz-se importante adotar medidas que minimizem invasões maliciosas ao ser adotado protocolo de acesso por meio do HTTPS *(Hypertext Transfer Protocol Secure)*, tais como: identificar a data de acesso dos usuários ao painel administrativo; reforçar a segurança para o uso de logins e senhas e, que esta senha contenha em sua formação letras maiúsculas e minúsculas, números e caracteres especiais.

Em vista disso, o desenvolvimento de um *software* com base nas normas de segurança é essencial para reduzir perdas de informações e economizar esforços e custos operacionais^32^. Desse modo, a preocupação com a segurança no WID^®^ partiu desde as etapas iniciais do ciclo de vida de *software* para que esse requisito fosse bem elaborado, atendendo aos quesitos de autenticação de dados, banco de dados, sistema para *backup*.

A manutenibilidade está relacionada à facilidade de modificação de um *software* durante toda sua evolução, desde correções do produto até adaptações dos requisitos. Por isso, torna-se desejável adotar boas práticas durante todo o desenvolvimento de *software* que beneficiem sua manutenção^33^. Desse modo, para o desenvolvimento do WID® foram utilizadas boas práticas de codificação, visando à adesão, uso e à facilidade de mantê-lo nas instituições de ensino. Diante disso, verificou-se que a manutenibilidade do *software* foi bem avaliada por parte dos *experts* técnicos. 

Fator relevante para a obtenção do sucesso de um *software* desenvolvido é a capacidade de adequação da tecnologia frente às mudanças que ocorrem com certa frequência no campo da saúde a partir do avanço do conhecimento, o que gera impacto positivo sob a manutenibilidade da tecnologia e adequação da mesma^34^. Desse modo, quanto menor o esforço/custo no ciclo de manutenção de *software*, maior tende a ser a qualidade do mesmo^35^.

Quanto à portabilidade, o WID^®^ foi desenvolvido com arquitetura do sistema com aplicações em *JavaScript* (interpretador de linguagem de programação de alto nível, do que se deseja colocar no *software* e potencial de migração), que estabelece ligação de programação com servidor e banco de dados. Além disso, o *software* é hospedado em um servidor na nuvem, na *Amazon Web Services* (AWS). 

Essas características são relevantes já que uma tecnologia só é considerada portável se ela pode ser executada em diferentes plataformas, envolvendo a modificação ou manutenção do *software* para que ele se adapte e execute em um novo ambiente. Trata-se do processo de mover um *software* de uma plataforma para outra. Quando o desenvolvimento da aplicação é realizado sob a linguagem de programação Java, há melhores indicadores de qualidade devido a sua portabilidade e dinamismo^35^.

As contribuições do *software* WID^®^ para o avanço do conhecimento científico para a enfermagem estão relacionadas à oferta de uma ferramenta de extensão e apoio confiável e válida para favorecer o processo de ensino-aprendizagem de estudantes e profissionais de saúde sobre o desenvolvimento do lactente.

Identifica-se como limitação a tecnologia não ter sido desenvolvida com direcionamento específico para públicos portadores de algum tipo de deficiência, podendo ser incluído este recurso nas próximas atualizações do *software*. Além disso, quanto ao aspecto metodológico, a coleta de dados se deu apenas de forma online, no entanto, acredita-se que poderia ter sido dada a opção da coleta presencial para os *experts* que moram na mesma região dos pesquisadores.

## Conclusão

O WID^®^ foi validado mediante as métricas de qualidade de *software* indicadas pela ISO/IEC 25010 e norma NBR ISO-IEC 14598-6, sendo considerado adequado em relação ao desempenho funcional e à qualidade técnica. Ressalta-se que o aperfeiçoamento desta tecnologia não se esgota apenas após as sugestões e avaliação dos *experts*. Este é um processo permanente que faz parte do ciclo de vida de um *software*. Portanto, novas ideias e atualizações podem emergir com a implementação e uso diário desta ferramenta no ensino da enfermagem.

Sugere-se a realização de estudos que abordem o WID^®^ com base nos efeitos da intervenção da tecnologia para a sua respectiva validação com o público-alvo de graduandos de enfermagem. Além disso, poderão ser desenvolvidos estudos para a aplicação do referido *software* em programas de treinamento de desenvolvimento de profissionais, sobretudo daqueles que atuam na atenção primária à saúde.

# Translation: **Evaluación de un **software* sobre el desarrollo del lactante que contribuye a la enseñanza y la capacitación profesional*** *

**DOI:** 10.1590/1518-8345.7248.4283

**Authors:** Wesley Soares de Melo, Hévila Ferreira Gomes Medeiros Braga, Maria Vera Lúcia Moreira Leitão Cardoso, Emanuella Silva Joventino Melo, Flávia Paula Magalhães Monteiro

**Affiliations:** 1 Universidade Federal do Ceará, Fortaleza, CE, Brasil.; 2 Universidade da Integração Internacional da Lusofonia Afro-Brasileira, Redenção, CE, Brasil.

**Keywords:** Enfermería Pediátrica, Desarrollo Infantil, Educación en Enfermería, Programas Informáticos, Tecnología Educacional, Estudio de Validación

## Abstract

**Objetivo:**

evaluar, junto con expertos, el desempeño funcional y la calidad técnica del *software* educativo *Wise Infant Development* ^*®*^ .

**Método:**

investigación metodológica que siguió el proceso de evaluación del *software* según las normas ISO/IEC 25010 y NBR ISO-IEC 14598-6. El desempeño funcional del *software* fue evaluado por un grupo de expertos en enfermería y la calidad técnica por expertos en tecnologías de la información. Para el análisis estadístico se utilizó el Índice de Validez de Contenido y la prueba Binomial.

**Resultados:**

la concordancia en los dos grupos de expertos fue superior al 70%, lo que indica que el *software* es adecuado y relevante para lo propuesto en todas las características evaluadas: adecuación funcional, confiabilidad, usabilidad, eficiencia en el desempeño, compatibilidad, seguridad, mantenibilidad y portabilidad. Los expertos realizaron sugerencias para mejorar la tecnología y fueron aceptadas.

**Conclusión:**

el *software Wise Infant Development* ^*®*^ fue bien evaluado por los expertos y puede contribuir a la enseñanza sobre el desarrollo del lactante, tanto en la carrera de enfermería como en la capacitación profesional.

## Destacados

**(1)** El *software* presenta calidad técnica y desempeño funcional satisfactorios.

**(2)** La tecnología es útil en el proceso de enseñanza-aprendizaje de los alumnos.

**(3)** El *software* se puede utilizar para formar profesionales en el área de la salud infantil.

## Introducción

Las tecnologías están cada vez más integradas en nuestra vida diaria, por lo que los profesionales, desde su formación, han buscado desarrollar y utilizar estrategias innovadoras. Por ello, las Tecnologías Digitales de la Información y la Comunicación (TDIC) pueden ser utilizadas como tecnologías educativas y son útiles como herramientas cognitivas que contribuyen a implementar un enfoque de enseñanza y aprendizaje híbrido o remoto^1^.

Durante el aislamiento social que impuso la pandemia de COVID-19, se intensificó la necesidad de utilizar la tecnología, especialmente en el ámbito educativo. Se ha convertido en una herramienta esencial para mejorar la enseñanza y el aprendizaje de las personas, así como para demostrar que el conocimiento puede ser accesible en cualquier lugar y en cualquier momento y adaptarse al ritmo de cada persona^2^ ^)-(^ ^3^.

En ese sentido, se observa que las TDIC también viene ocupando espacios en la formación de enfermería, tanto en las carreras de grado como en los cursos de posgrado, así como también en la capacitación profesional. El uso de TDIC ha demostrado que tiene muchos beneficios para la enseñanza-aprendizaje, como la posibilidad de personalizar la enseñanza, optimizar procesos, reducir las tasas de deserción, además, las innovaciones tecnológicas contribuyen al desarrollo de habilidades clínicas y contribuyen al proceso de toma de decisiones en salud^4^ ^)-(^ ^5^.

En el contexto de la salud del niño, los enfermeros deben evaluar rigurosamente el desarrollo infantil, especialmente durante las consultas de puericultura, tanto para hacer un seguimiento adecuado como para realizar intervenciones tempranas, si se identifica algún retraso. Para eso se desarrolló el *Wise Infant Development* (WID^®^), un *software* educativo, alojado en un servidor *web*, desarrollado en lenguaje de programación *JavaScript* y a la luz de la Teoría de la Epistemología Genética de Piaget desde una perspectiva Cognitivo Constructivista^6^.

Esta tecnología puede contribuir al proceso de enseñanza-aprendizaje de los futuros enfermeros y a la capacitación profesional sobre el desarrollo infantil del lactante, para mejorar la atención de la salud infantil^7^. El enfermero es responsable de brindarle atención y apoyo a los niños y sus familias, identificando e interviniendo ante las necesidades y vulnerabilidades^8^ ^)-(^ ^9^.

Aún hay profesionales que no evalúan adecuadamente el desarrollo neuropsicomotor o que confunden esa evaluación con el estado general del niño y las medidas de crecimiento, sin tener en cuenta las acciones que incluyen la evaluación de los hitos y del riesgo de retraso en el desarrollo para la edad. Por ende, comprender adecuadamente la evaluación del desarrollo infantil es un criterio decisivo para brindar atención cualificada y realizar intervenciones oportunas y específicas^10^.

Por lo tanto, las estrategias de capacitación deben brindarles a los estudiantes y profesionales experiencias que los impulsen a reflexionar y actuar en el contexto del cuidado de la salud. El aprendizaje mediado por computadora puede sumarles más interactividad a los enfoques convencionales de educación. Por ello, la inclusión de un *software* en el proceso de enseñanza y aprendizaje puede facilitar la adquisición de conocimientos y suplir las deficiencias de los enfoques tradicionales de evaluación del desarrollo infantil^7^.

Sin embargo, cualquier tecnología educativa debe pasar por un riguroso proceso de evaluación antes de aplicarla al público objetivo. Por eso, es necesario que el proceso de evaluación de productos garantice soporte técnico, pedagógico y metodológico, además de recursos y funciones satisfactorias, así como contenidos suficientes y comprensibles para el objetivo para el que fue desarrollada^11^.

Por lo tanto, evaluar la calidad del WID^®^ es un procedimiento importante para garantizar que el *software* realice sus funciones correctamente y esté aprobado según las normas técnicas de credibilidad.

La enseñanza impartida a través de esta tecnología podría ser un momento privilegiado de construcción del saber con el fin de estimular la consolidación de un cuerpo de conocimiento sobre el tema en cuestión. Por lo tanto, el presente estudio tuvo como objetivo evaluar, junto con expertos, el desempeño funcional y la calidad técnica del *Wise Infant Development*-WID^®^, un *software* educativo que contribuye a la enseñanza del desarrollo del lactante. 

## Método

### Diseño del estudio

Estudio de evaluación metodológica y tecnológica. La tecnología evaluada es el *software* educativo denominado *Wise Infant Development*-WID^®^, que está registrado en el Instituto Nacional de Propiedad Industrial-INPI y, por lo tanto, cuenta con garantía de validez en Brasil y en los otros 176 países que forman parte del Convenio de Berna (1886). El estudio siguió las directrices SQUIRE.

### Lugar y período de recolección de datos

El *software* fue desarrollado de enero de 2019 a febrero de 2020, y la etapa de evaluación con expertos se realizó de febrero a marzo de 2020, en una Institución de Educación Superior (IES), ubicada en el municipio de Redenção, Ceará, Brasil. 

### Participantes y criterios de selección

La selección del panel de expertos siguió las directrices de la norma NBR ISO/IEC 14598-6, que es una norma específica que indica el número de expertos y recomienda la inclusión de al menos ocho miembros en cada grupo de evaluadores, para garantizar la representatividad de la categoría de los usuarios del *software* ^12^. Por lo tanto, el estudio contó con la participación de dos grupos de expertos: 1. Enfermeros que trabajan en las áreas de salud infantil, desarrollo infantil y tecnologías; y 2. Profesionales que trabajan en el área de tecnologías de la información (TI) especialmente en desarrollo/validación de *software*.

Para seleccionar a los enfermeros expertos se consideraron los siguientes criterios adaptados: tener tesis y/o disertación sobre el tema Desarrollo Infantil del Lactante; contar con al menos un año de experiencia docente en materias del área de Salud Infantil; tener especialización en Enfermería Pediátrica con proyecto de finalización de curso enfocado en el desarrollo infantil; tener una práctica clínica de al menos un año en el área de Salud Infantil; haber publicado investigaciones/artículos sobre Salud Infantil con contenido relevante para el área en cuestión^13^. Cabe aclarar que la adaptación mencionada se refiere a enfocar los criterios en las áreas de interés del estudio.

Para seleccionar a los expertos en TI, los criterios adaptados establecidos fueron: tener una tesis o disertación en el tema de ingeniería de *software* y/o análisis de sistemas; tener especialización en ingeniería de *software* o un área relacionada; tener producción científica en materia de ingeniería de *software* y/o análisis de sistemas; tener desarrollo de *software*; tener experiencia profesional de al menos un año en análisis de sistemas y/o desarrollo de *software* ^13^. 

Los expertos fueron identificados a partir de los CV de la *Plataforma Lattes* mediante la aplicación de filtros, como los relacionados con la formación académica, la actuación profesional, las actividades de orientación y la presencia en el directorio de grupos de investigación para ambos grupos de expertos. Se priorizaron profesionales que demostraron experiencia significativa en las áreas específicas mencionadas^13^.

En este proceso también se utilizó la técnica de bola de nieve para identificar potenciales expertos. Cuando los autores contactaron por correo electrónico a los profesionales seleccionados a través de la *Plataforma Lattes*, los invitaron a participar en el estudio y les solicitaron que recomendaran a otros expertos que podrían colaborar en la evaluación.

Todo el contacto en la investigación se realizó por correo electrónico. Se envió una carta de invitación a la dirección de correo electrónico de los expertos. En total fueron invitados 70 profesionales, de los cuales 19 aceptaron participar en el estudio. Después de que aceptaron, se les envió el Formulario de Consentimiento Libre e Informado (FCLI) que contiene la firma digital del investigador responsable y se les solicitó que lo devolvieran con la firma digital o escaneada del participante. Los evaluadores tuvieron un plazo de 30 días para devolver sus evaluaciones, tiempo durante el cual el *software* era de acceso libre para los expertos. Tres participantes fueron excluidos porque no se contactaron con los investigadores. 

### Instrumentos utilizados para recopilar información

El proceso de evaluación se operacionalizó según la norma internacional ISO/IEC 25010 (*System and Software engineering-System and software Quality Requirements and Evaluation-SQuaRE-System and software quality models*), que considera las características de calidad del producto de *software* ^14^ que se presentan en la Figura 1.

**Figura 1:** Características de la norma ISO/IEC 25010 del modelo de calidad del *software*

**Calidad del sistema/Producto de *Software***		
Característica	Subcaracterística	Definición
**Adecuación funcional**	Integridad funcional; Corrección funcional; Aptitud funcional.	Se relaciona con la necesidad de que las funcionalidades del *software* cumplan con lo solicitado en los requisitos.
**Confiabilidad**	Madurez; Tolerancia a fallos; Recuperabilidad; Disponibilidad.	Se relaciona con la capacidad del *software* para mantener su nivel de desempeño en condiciones establecidas durante un período de tiempo. Esta característica se percibe cuando el *software*, bajo ciertas condiciones (ejemplo: escasez de recursos), puede realizar sus funcionalidades de manera confiable.
**Usabilidad**	Reconocimiento de adecuación; Aprehensibilidad; Protección contra errores; Operabilidad; Estética de la interfaz de usuario; Accesibilidad.	Se relaciona con el esfuerzo necesario para utilizar el *software*, así como con la opinión individual sobre el uso, por parte de un grupo de usuarios. Indica que el *software* puede ser utilizado por usuarios específicos con determinados niveles de efectividad, eficiencia y satisfacción.
**Eficiencia en el desempeño**	Tiempo; Recursos; Capacidad	Característica que relaciona el nivel de desempeño del *software* y la cantidad de los recursos utilizados, bajo condiciones establecidas.
**Compatibilidad**	Coexistencia; Interoperabilidad.	Se relaciona con la calidad del producto, sistema o componente, intercambiar información con otros productos, sistemas o componentes, y/o realizar las funciones necesarias, cuando comparte el mismo entorno de *hardware* o *software*. El objetivo es que el *software* pueda intercambiar información con otros sistemas en el mismo entorno operativo.
**Seguridad**	Confidencialidad; Integridad; No repudio; Responsabilidad; Autenticación.	Se relaciona con la protección de la información y los datos y el control del nivel de acceso de personas, productos o sistemas según los tipos y niveles de autorización. Se observa cuando el *software* protege su información y los datos según los niveles de autorización establecidos.
**Mantenibilidad**	Analizabilidad; Modificabilidad; Modularidad; Reutilización; Testeabilidad.	Se relaciona con el esfuerzo necesario para realizarle modificaciones específicas al *software*.
**Portabilidad**	Adaptabilidad; Capacidad de navegación; Reemplazabilidad.	Se relaciona con la capacidad del *software* para ser transferido de un entorno a otro. Verifica que el *software* se pueda transferir a otro entorno operativo definido en sus requisitos de manera eficiente y efectiva.

### Recolección de datos

El *software* está organizado en las siguientes funciones: funciones básicas, funciones prueba previa y prueba posterior, pantalla principal del *software*, información sobre la tecnología, módulos de enseñanza, pruebas, certificado, preguntas frecuentes, editar perfil y panel de administración del *software*.

En cuanto a las funciones básicas del *software*, tiene funciones esenciales, como: la pantalla de inicio para acceder a la tecnología basada en la identificación del usuario y las opciones: “Restablecer contraseña” y “Registrar nuevo usuario”. Después de iniciar sesión, los usuarios son dirigidos automáticamente a una pantalla de bienvenida seguida de información sobre una prueba previa, que consta de 16 preguntas de opción múltiple, que se utilizan para evaluar el conocimiento previo de los estudiantes sobre el desarrollo infantil. Esta estrategia tiene como objetivo medir la efectividad del *software* como herramienta educativa. Luego de completar la prueba previa, una pantalla informa el final de esta etapa y el número total de respuestas correctas, sin revelar cuáles fueron las preguntas respondidas correctamente para evitar sesgos de memoria en la prueba posterior.

Luego de completar la prueba previa, los estudiantes son redirigidos a la pantalla principal, la cual muestra la identificación del estudiante y les ofrece las siguientes opciones de navegación: información sobre la tecnología, módulos de enseñanza, pruebas, certificado, preguntas frecuentes, editar perfil y salir. El ítem información sobre la tecnología proporciona una descripción de qué es el *software*, su objetivo y contribución a la práctica de enfermería.

Los módulos de enseñanza, se estructuraron de la siguiente manera: Módulo I - Introducción (4 clases; 4 pantallas); Módulo II - Desarrollo físico (15 clases; 42 pantallas); Módulo III - Desarrollo cognitivo (6 clases; 14 pantallas); Módulo IV - Desarrollo psicosocial (5 clases; 5 pantallas); Módulo V - Desarrollo infantil en Brasil (6 clases; 12 pantallas). El contenido de los módulos se presenta a través de textos y diagramas de flujo que se pueden ampliar para optimizar la visualización, y además cuentan con acceso a fotografías y videos obtenidos del sitio *web* del Ministerio de Salud de Brasil. Se puede acceder a ellos en la plataforma WID^®^ para mejorar aún más el proceso de aprendizaje. 

Todo el contenido está organizado en “Lecciones” numeradas, que indica claramente cómo localizar el contenido y que la distribución es efectiva. Este enfoque facilita la memorización y la toma de notas durante el uso del *software*, la intención es que la presentación y el formato de uso sean didácticos.

Cabe señalar que el contenido del *software* fue creado a partir de una revisión de la literatura realizada por dos revisores en las siguientes bases de datos: Literatura Latinoamericana y del Caribe en Ciencias de la Salud (LILACS), *Medical Literature Analysis and Retrieval System Online* (MEDLINE), *Scientific Electronic Library Online* (SciELO) y *Portal da Coordenação de Aperfeiçoamento de Pessoal de Nível Superior no Brasil* (Portal CAPES - plataforma que reúne producciones científicas brasileñas e internacionales). Se utilizaron los términos MeSH/DeCS controlados “*infant*” y “*infant development*” y los termos no controlados “*physical development*”, “*psychosocial development*” y “*cognitive development*” combinados por el operador booleano “AND” ^(^ ^7^.

Los títulos y resúmenes fueron leídos y seleccionados para revisión del texto completo. Fueron incluidos artículos científicos disponibles electrónicamente con texto completo, en las bases de datos elegidas, publicados en portugués o inglés, en los últimos 5 años. También se consideraron manuales, libros y publicaciones oficiales del Ministerio de Salud. Se excluyeron los textos duplicados y los estudios que no abordaban el desarrollo infantil^7^.

La revisión incluyó 22 artículos, tres manuales, siete libros y una publicación oficial del Ministerio de Salud de Brasil^7^.

Después de completar todos los módulos, el *software* redirige a los estudiantes a la pantalla prueba posterior. Cabe destacar que las preguntas presentes en la prueba posterior son idénticas a las de prueba previa, con el objetivo de permitirles realizar un análisis comparativo y contribuir a que evalúen los conocimientos adquiridos. Se cambia la disposición de las preguntas de la prueba posterior con respecto a la prueba previa, con el fin de evitar la memorización.

En la sección denominada “Pruebas”, los usuarios pueden comprobar el número de preguntas respondidas correctamente tanto en la prueba previa como en la prueba posterior, una vez que completaron todos los módulos de enseñanza. Caso contrario, el *software* les notifica a los usuarios que es imprescindible completar todos los módulos para poder comenzar la prueba posterior.

Con respecto al certificado, el usuario tiene acceso al certificado de finalización del curso realizado a través del *software*, si ha completado todos los módulos y la prueba posterior. El certificado acredita la participación en el curso, contiene el nombre completo del estudiante, la fecha de finalización, la carga horaria, detalles sobre los módulos de enseñanza, el logotipo del *software* y la institución educativa responsable del curso. Además, la sección Preguntas Frecuentes muestra respuestas y apoyo adecuado para ayudar a los usuarios con cualquier pregunta sobre el *software*.

El perfil del usuario contiene la información proporcionada durante el registro y estos datos se pueden editar en cualquier momento utilizando la función “Editar perfil”. Además, hay un panel de administración designado para realizar un seguimiento. Esto le permite al tutor o profesor supervisar el progreso y gestionar las actividades de los estudiantes en el *software*. El panel proporciona datos de registro que incluyen el desempeño en las pruebas, el progreso en los módulos de enseñanza y facilita una mediación pedagógica efectiva.

Para recolectar los datos de los participantes se utilizó un instrumento que contempla todas las características de la norma ISO/IEC 25010, el cual fue traducido y adaptado para la evaluación de *software* en investigaciones de esta naturaleza, y los enfermeros expertos evalúan seis características: adecuación funcional, confiabilidad, usabilidad, eficiencia en el desempeño, compatibilidad y seguridad. Los expertos en el área de tecnologías de la información evaluaron las ocho características mencionadas, más las características de mantenibilidad y portabilidad, dado que cuentan con datos técnicos específicos para este grupo de expertos.

A cada ítem evaluado se le asignó un concepto ordenado en una escala ordinal de cinco puntos con las siguientes clasificaciones: 1 - Nada apropiado; 2 - Poco apropiado; 3 - Moderadamente apropiado; 4 - Muy apropiado; 5 - Completamente apropiado^15^. Los investigadores enviaron por correo electrónico a los expertos los siguientes archivos PDF: mapas de interacción y navegación del *software* con explicaciones sobre la arquitectura de la tecnología e instrucciones de uso, así como el instrumento de evaluación. Posteriormente se recibió el archivo cumplimentado, también de forma *online*.

El juicio del proceso de evaluación de las características/subcaracterísticas analizadas se realizó según la norma NBR ISO-IEC 14598-6^11^ adaptada por Sperandio^16^, en la que el autor menciona que el valor mínimo de respuestas marcadas como apropiadas debe ser del 70% (muy apropiado o completamente apropiado) para que las características/subcaracterísticas se consideren adecuadas. 

### Procesamiento y análisis de datos

Los datos de la investigación fueron analizados utilizando el IBM *Statistical Package for the Social Sciences* (SPSS), versión 26.0. Se utilizó el índice de validez de contenido (IVC) y la prueba binomial con una proporción de 0,70, que consiste en un valor de concordancia esperado igual o mayor al 70% con un nivel de significación del 5% (alfa = 0,05)^17^.

### Aspectos éticos

El estudio fue aprobado por el Comité de Ética en Investigación de la *Universidade da Integração Internacional da Lusofonia Afro-Brasileira* (UNILAB), bajo dictamen n.º 3.465.662 y certificado de apreciación n.º 08328319.5.0000.5576.

## Resultados

La muestra estuvo compuesta por 16 evaluadores, ocho en cada grupo. En el grupo de enfermeros, la mayoría de los participantes eran del sexo femenino (n=7), de los cuales: cuatro tenían doctorado, tres maestrías y uno posdoctorado. Tenían una edad promedio de 35,6 (±6,36) años y un tiempo medio de actuación en el área de 13,1 (±6,79) años, el área de actividad predominante era docencia e investigación (n=6). Este grupo estuvo conformado por expertos de las regiones Nordeste, Centro-Oeste y Sur de Brasil, que trabajan en diferentes IES públicas y privadas. En el grupo de los profesionales de TI predominó el sexo masculino, tenían una edad promedio de 31,3 (± 4,20) años. Cuatro cuentan con maestría, tres con doctorado y uno con especialización. El tiempo medio de trabajo en el área era de 6,7 (±2,18) años, las áreas de actividad predominantes eran docencia (37,5%) y docencia/investigación (37,5%), con tres expertos en cada. Todos los expertos eran de la región Nordeste de Brasil y trabajaban en diferentes IES públicas y privadas.

Los resultados de la evaluación de ambos grupos de expertos sobre las características/subcaracterísticas de la norma ISO/IEC 25010 del modelo de calidad indican que el *software* es adecuado y relevante para lo propuesto, la mayoría de las preguntas obtuvo un 70% de concordancia. Se observa que sólo la subcaracterística accesibilidad no alcanzó el porcentaje adecuado de concordancia (>70%) para ser considerada de calidad, por lo que fue necesario realizar correcciones antes de aplicarla al público objetivo, como se puede ver en la Tabla 1.

**Tabla 1:** Evaluación de los expertos según la norma ISO/IEC 25010 (n = 16). Redenção, CE, Brasil, 2020

Características	Enfermeros expertos			Expertos en TI*		
	IVC^†^	Binomial	*p-valor*	IVC^†^	Binomial	*p-valor*
**Adecuación funcional**						
Integridad funcional	0,87	0,90	0,001	0,87	0,90	0,001
Corrección funcional	0,87	0,90	0,001	0,87	0,90	0,001
Aptitud funcional	1,00	1,00	0,058	1,00	1,00	0,058
**Confiabilidad**						
Madurez	0,75	0,80	0,011	0,75	0,80	0,011
Tolerancia a fallos	1,00	1,00	0,058	0,75	0,80	0,011
Recuperabilidad	1,00	1,00	0,058	0,87	0,90	0,001
Disponibilidad	1,00	1,00	0,058	0,87	0,90	0,001
**Usabilidad**						
Reconocimiento de adecuación	1,00	1,00	0,058	0,87	0,90	0,001
Aprehensibilidad	1,00	1,00	0,058	1,00	1,00	0,058
Operabilidad	0,87	0,90	0,001	0,87	0,90	0,001
Accesibilidad	0,00	0,00	0,058	0,12	0,10	0,255
Protección contra errores	1,00	1,00	0,058	0,87	0,90	0,001
Estética de interface del usuario	1,00	1,00	0,058	1,00	1,00	0,058
**Eficiencia en el desempeño**						
Tiempo	1,00	1,00	0,058	0,87	0,90	0,001
Recursos	1,00	1,00	0,058	0,87	0,90	0,001
Capacidad	1,00	1,00	0,058	1,00	1,00	0,058
**Compatibilidad**						
Interoperabilidad	0,87	0,90	0,001	1,00	1,00	0,058
Coexistencia	1,00	1,00	0,058	0,87	0,90	0,001
**Seguridad**						
Confidencialidad	1,00	1,00	0,058	0,75	0,80	0,011
Integridad	1,00	1,00	0,058	0,87	0,90	0,001
No repudio	0,87	0,90	0,001	0,87	0,90	0,001
Responsabilidad	1,00	1,00	0,058	1,00	1,00	0,058
Autenticación	0,87	0,90	0,001	0,87	0,90	0,001
**Mantenibilidad**						
Analizabilidad	-	-	-	0,75	0,80	0,011
Modificabilidad	-	-	-	1,00	1,00	0,058
Modularidad	-	-	-	0,87	0,90	0,001
Testeabilidad	-	-	-	0,75	0,80	0,011
Reutilización	-	-	-	1,00	1,00	0,058
Portabilidad						
Adaptabilidad	-	-	-	1,00	1,00	0,058
Capacidad de navegación	-	-	-	1,00	1,00	0,058
Reemplazabilidad	-	-	-	1,00	1,00	0,058

*TI = Tecnología de la información; ^†^IVC = Índice de validez de contenido

Los expertos solo hicieron sugerencias sobre cinco características en cuanto al desempeño funcional y calidad técnica del WID^®^, las cuales fueron aceptadas en el proceso de mejora de la tecnología evaluada, como se muestra en la Figura 2. 

**Figura 2:** Sugerencias de los expertos sobre la evaluación del desempeño funcional y la calidad técnica del WID^®^

Enfermeros expertos	Expertos en TI*
**Adecuación funcional**	
En el contenido sobre el desarrollo físico, abordar los pliegues de la piel del lactante. Algunos autores de renombre consideran importante llevar a cabo esta medida. En el desarrollo cognitivo, especificar los tipos de lenguaje que tiene el lactante (receptivo y no receptivo) y qué se espera en cada etapa.	Puede ser interesante incluir vídeos y actividades para mostrar el progreso del alumno. Porque, como solo es contenido, el alumno puede recorrer todas las páginas sin leer lo que hay en el contenido.
Proporcionar texto más interactivo para el lector. Hacer preguntas y fomentar la reflexión, no sólo en los ejercicios de fijación, sino a lo largo del texto.	El panel de administración podría tener búsqueda por usuarios y paginación. A medida que crece el número de usuarios, la tabla del panel puede resultar difícil de mantener.
Presentar dibujos para enfatizar cómo se deben realizar las evaluaciones, además de vídeos. Mostrar en los dibujos los puntos importantes a los que hay que prestarles atención y que la figura no aborda, por ejemplo, cómo medir la circunferencia de la cabeza.	Debería haber más mecanismos para evaluar si el estudiante realmente leyó el contenido y si fue capaz de asimilar lo enseñado.
Permitir que los alumnos accedan a las preguntas de la prueba posterior en las que su respuesta fue incorrecta, así como a la hoja de respuestas correctas, con el fin de fomentar el aprendizaje.	Reparar el progreso de los módulos en la pantalla de inicio que a veces no se mostraba e impedía que realizaran la prueba posterior.
	Solucionar los problemas de navegación entre módulos. Cuando el software se colgaba, era necesario actualizar la página y, por lo tanto, volver a conectarse.
**Confiabilidad**	
Solucionar el pequeño error que hace que cada vez que completamos un módulo, el *software* nos redirige a la prueba posterior.	Solucionar las fallas en el botón “siguiente clase” entre los módulos didácticos II y III.
Mejorar la velocidad, lentitud para cargar las siguientes clases. Después de conectarme nuevamente, funcionó a mejor velocidad.	Corregir errores sobre la realización de la prueba posterior. Apareció el mensaje “estudiante no encontrado”.
Solucionar pequeñas fallas: el *software* se colgó y cuando actualicé la página, tuve que iniciar sesión nuevamente. Pero cuando me conecté nuevamente, no tuve que rehacer la prueba previa.	Solucionar los errores durante la ejecución de los módulos para que no sea necesario volver a la pantalla de inicio para continuar con el proceso de aprendizaje.
	Hay momentos en los que el *software* no puede guardar el progreso de las actividades. Realizar corrección técnica.
Solucionar los errores en la ejecución de la prueba posterior.	Cuando pierdes una conexión durante una clase y haces clic en la siguiente clase, el botón desaparece y sigue cargando infinitamente. Lo ideal sería mostrarle al usuario un mensaje y volver al botón siguiente clase.
**Usabilidad**	
Mayor interacción en cada módulo, con casos clínicos, fotografías, videos, ejercicios de reflexión.	Al final de cada módulo, incluir actividades para evaluar el contenido aprendido.
Reducir la escritura y crear esquemas más lúdicos.	Contar con recursos como *Anno*, *aSimpleTour*, *Bootstrap.js*, entre otros, que explican las principales secciones del sistema.
	El panel de administración podría tener mayor control sobre los usuarios (eliminar usuario, cambiar contraseña, actualizar datos, bloquear usuario, etc.).
	Agregar funciones como aumentar/disminuir fuente, cambiar contraste, teclas de acceso, entre otras.
Aumentar la letra y el espaciado. Añadir más figuras como ejemplo.	Agregar “lupas” que funcionen solo en el *software* y “caja de sonido” para que se pueda leer el texto.
	Mejorar la capacidad de respuesta en el *layout*.
	Informar el error de que completar el campo es obligatorio.
**Eficiencia en el desempeño**	
Ampliar el vídeo. Es muy pequeño en la esquina superior derecha de la pantalla.	Incrementar el uso de recursos de vídeo y audio, son elementos esenciales en una plataforma de enseñanza.
**Seguridad**	
Identificar fecha de acceso, además del autor, en el panel de administración.	No se utiliza el protocolo HTTPS^†^. Protocolo esencial para el uso de *logins* y contraseñas. Sugiero cambiar.
	Sugerirle al usuario que ingrese una contraseña segura, informarle el número mínimo de caracteres, letras, al menos una letra mayúscula, números y caracteres especiales.
	Mejorar la solicitud de inicio de sesión. Este se envía en el cuerpo de la solicitud, y es accesible para cualquier *software* malicioso.
	Identificar registros con fecha y hora de acceso.

*TI = Tecnología de la información; ^†^HTTPS = *Hyper Text Transfer Protocol Secure*

## Discusión

Todo *software* educativo debe pasar por una evaluación previa antes de ser implementado en el contexto educativo. Esta evaluación tiene como objetivo identificar si la tecnología en cuestión tiene características satisfactorias con respecto a los aspectos pedagógicos, que incluyen la calidad del contenido propuesto, la interfaz centrada en la usabilidad, los elementos relacionados con la calidad técnica y funcional propiamente dicha^11^. En este caso, se observó que el WID^®^ logró resultados satisfactorios sobre su desempeño funcional y calidad técnica en la evaluación de los expertos.

Un estudio realizado sobre el análisis de los requisitos de calidad en tecnologías duras en el contexto de la educación en salud identificó que uno de los principales puntos que afectan la calidad del *software* es la adecuación funcional^18^. Sobre esa característica, los expertos solicitaron mayores implementaciones enfocadas en el contenido y que se aborde de forma más atractiva y dinámica, a través de dibujos, imágenes, que incluya más actividades en los módulos de enseñanza y que se solucionen problemas generales sobre la tecnología.

Considerando que el desarrollo infantil implica una serie de transformaciones progresivas y complejas, influenciadas por factores internos, externos y el entorno de cuidado, es crucial comprender y destacar en las tecnologías de enseñanza las características únicas del desarrollo del lactante para promover su salud y que alcance sus habilidades generales. Por eso, es fundamental emplear tecnologías que puedan ayudar a los profesionales y estudiantes del área de la salud a monitorear el desarrollo de los niños^19^ ^)-(^ ^20^. Asimismo, los recursos educativos que incorporan elementos audiovisuales pueden captar la atención del espectador de forma multisensorial, ofrecerle interactividad y promover una comprensión más efectiva del contenido, que enriquezca la experiencia de aprendizaje^21^. 

El análisis de confiabilidad es fundamental para que podamos actuar preventivamente ante posibles fallas y garantizar una mayor disponibilidad de la tecnología para su uso^22^ ^)-(^ ^23^. En este sentido, los expertos, especialmente los del área de tecnologías de la información, aportaron importantes sugerencias para solucionar los problemas del WID^®^ y mejorarlo. Esto implica que el análisis de confiabilidad es una práctica fundamental para asegurar que los sistemas y equipos permanezcan operativos y confiables, minimizando las interrupciones y los impactos en el mercado.

En esta tecnología es necesario llevar a cabo reparaciones técnicas para ejecutar las clases de los módulos de enseñanza, mejorar la velocidad de navegación y carga de contenidos, solucionar posibles errores relacionados con el registro de usuarios y mejorar el almacenamiento y el progreso de las actividades que realizaron los estudiantes.

La usabilidad del WID^®^ demostró ser aceptable según la evaluación de los expertos. Este hecho nos permite afirmar que el *software* es agradable y satisface las necesidades para las que está destinado. Las pruebas de usabilidad se vuelven cada vez más relevantes en el proceso de evaluación, dado que buscan mejorar la experiencia del usuario^24^ ^)-(^ ^25^. Por ello, es muy importante que participen expertos, especialmente los del área de tecnologías de la información, que dominan esta característica y, por eso, realizaron inspecciones y pruebas minuciosas para garantizar un buen producto, con el objetivo de asegurar la calidad y aceptación del WID^®^.

Otras consideraciones necesarias para la usabilidad en general, tales como: necesidad de una mayor interacción en cada módulo con casos clínicos, fotografías, videos, esquemas lúdicos y ejercicios de reflexión; incluir actividades al final de cada módulo para evaluar los contenidos aprendidos, además de las reparaciones técnicas mencionadas, coinciden con las sugerencias dadas para lograr la adecuación funcional, ya que ambas se relacionan a medida que el *software* cumple con lo propuesto, asegurándole, además, una buena experiencia al usuario.

Los recursos mencionados, como *Anno, aSimpleTour, Bootstrap.js*, fueron sugeridos con la intención de brindar funciones que trataran de explicar las principales secciones del sistema y cómo navegar por el *software*. Además, otro punto que requiere atención es la capacidad de respuesta del *layout* del WID^®^, que se relaciona con la capacidad de la tecnología para adaptarse a diferentes tipos de entornos y pantallas, como, por ejemplo, *smartphones* y *tablets*.

Aunque el WID^®^ fue bien evaluado en términos de usabilidad, la subcaracterística accesibilidad del WID^®^ para personas con discapacidad visual se consideró inapropiada y recibió sugerencias importantes de ambos grupos de expertos, que incluyen: agregar recursos como aumentar/disminuir la fuente, cambiar el contraste, teclas de acceso, aumentar la letra y el espaciado, agregar “lupas” que funcionan solo en el *software* y “caja de sonido” para que haya audiodescripción del contenido. Sin embargo, cabe señalar que esta tecnología fue diseñada para ser utilizada por personas con baja agudeza visual (discapacidad visual parcial), que les permite utilizar la función zoom del navegador, que aumentará automáticamente el tamaño de la fuente de las letras e imágenes presentes en el *software*, facilitando la legibilidad y navegación.

La característica eficiencia en el desempeño recibió una evaluación satisfactoria por parte de los expertos. El criterio de agilidad es considerado de alta importancia y singularidad al momento de evaluar el desempeño del *software* en función de la eficiencia y/o efectividad de las acciones propuestas en la tecnología^26^ ^)-(^ ^27^. Un estudio realizado sobre evaluación y comparación entre *software* destaca que la eficiencia del desempeño es un punto crucial para elegir el *software* e implementarlo en el ambiente para el cual fue propuesto^28^.

La evaluación de los expertos fue fundamental, dado que, además de cuantificar la concordancia, identificaron dificultades en el desempeño de la tecnología y sugirieron acciones para mejorar el WID^®^, que fueron aceptadas y resueltas en la versión final del *software*, a saber: capacidad de ampliar vídeos e implementar recursos multimedia, dado que son elementos esenciales en una plataforma de enseñanza. Esta mejora es necesaria, ya que el proceso de aprendizaje se ha vuelto más dinámico y flexible con la llegada de tecnologías que les proporcionan a los estudiantes diferentes metodologías, recursos y métodos^29^.

Dependiendo de la complejidad de la tecnología desarrollada, existen varios desafíos a superar para lograr la compatibilidad total en los sistemas en el campo de la salud, y es fundamental que los profesionales de las áreas de tecnología/desarrollo de *software* y atención médica trabajen juntos para mejorar esa característica^30^. Por lo tanto, las sugerencias de los expertos contribuyen a mejorar pequeñas fallas entre los módulos del WID^®^ y del sistema en red, que, si bien no tuvieron un gran impacto en la tecnología, también repercuten en la compatibilidad.

Los expertos realizaron importantes sugerencias para mejorar la seguridad del *software*, que fueron debidamente aceptadas. La calidad de la característica seguridad actúa sobre la integridad de la información, es decir, sobre la prevención de ataques a los datos para garantizar que los sistemas se restablezcan y que se garantice el acceso seguro a la información incluso cuando los ataques al sistema informático tengan éxito^31^. Por ende, es importante adoptar medidas para minimizar la entrada de *software* maliciosos usando un protocolo de acceso a través de HTTPS (*Hypertext Transfer Protocol Secure*), tales como: identificar la fecha en la que los usuarios acceden al panel de administración; reforzar la seguridad para el uso de *logins* y contraseñas y que la contraseña contenga letras mayúsculas y minúsculas, números y caracteres especiales.

Por lo tanto, es fundamental desarrollar un *software* basado en estándares de seguridad para reducir las pérdidas de información y ahorrar esfuerzos y costos operativos^32^. Por ello, la preocupación por la seguridad del WID^®^ estuvo presente desde las etapas iniciales del ciclo de vida del *software,* para que este requisito sea bien elaborado y cumpla con los ítems autenticación de datos, base de datos, sistema de *backup*.

La mantenibilidad se relaciona con la facilidad para modificar el *software* a lo largo de su evolución, desde correcciones de productos hasta adaptaciones de requisitos. Por eso, conviene adoptar buenas prácticas en todo el desarrollo del *software* que favorezcan el mantenimiento^33^. Por lo tanto, para desarrollar el WID^®^ se utilizaron buenas prácticas de codificación, para lograr adherencia, uso y facilidad de mantenimiento del mismo en las instituciones educativas. Por ello, se comprobó que la mantenibilidad del *software* fue bien evaluada por los expertos técnicos.

Un factor relevante para lograr el éxito de un *software* desarrollado es la capacidad de adaptar la tecnología a los cambios que se presentan con cierta frecuencia en el campo de la salud debido al avance del conocimiento, lo que tiene un impacto positivo en la mantenibilidad de la tecnología y la adecuación de la misma^34^. Por ende, cuanto menor es el esfuerzo/coste en el ciclo de mantenimiento del *software*, mayor tiende a ser su calidad^35^.

En cuanto a la portabilidad, el WID^®^ fue desarrollado con una arquitectura de sistema con aplicaciones en *JavaScript* (intérprete de lenguaje de programación de alto nivel, de lo que se quiere poner en el *software* y potencial de migración), que establece una conexión de programación con el servidor y la base de datos. Además, el *software* está alojado en un servidor en la nube, en *Amazon Web Services* (AWS).

Estas características son relevantes, ya que una tecnología sólo se considera portátil si puede ejecutarse en diferentes plataformas, lo que implica la modificación o el mantenimiento del *software* para que se adapte y corra en un nuevo entorno. Es el proceso de mover *software* de una plataforma a otra. Cuando el desarrollo de aplicaciones se realiza utilizando el lenguaje de programación Java, existen mejores indicadores de calidad debido a su portabilidad y dinamismo^35^.

Las contribuciones del *software* WID^®^ al avance del conocimiento científico para la enfermería se relacionan con aportar una herramienta de extensión y apoyo confiable y válida que favorezca el proceso de enseñanza-aprendizaje de estudiantes y profesionales de la salud sobre el desarrollo del lactante.

Se considera una limitación que la tecnología no fuera desarrollada específicamente para personas con algún tipo de discapacidad, y esa característica puede incluirse en futuras actualizaciones del *software*. Además, en cuanto al aspecto metodológico, la recolección de datos se realizó únicamente *online*, sin embargo, se cree que a los expertos que viven en la misma región que los investigadores se les podría haber dado la opción de responder los instrumentos de forma presencial.

## Conclusión

El WID^®^ fue validado utilizando las métricas de calidad del *software* que indica la norma ISO/IEC 25010 y la norma NBR ISO-IEC 14598-6, y se consideró adecuado con respecto al desempeño funcional y la calidad técnica. Cabe señalar que la mejora de esta tecnología no termina únicamente con las sugerencias y evaluaciones de los expertos. Este es un proceso permanente que forma parte del ciclo de vida del *software*. Por lo tanto, pueden surgir nuevas ideas y actualizaciones con la implementación y el uso diario de esta herramienta en la educación de enfermería.

Se sugiere realizar estudios que aborden el WID^®^ en función de los efectos de la intervención tecnológica para su respectiva validación con el público objetivo, que son los estudiantes de enfermería. Además, se pueden desarrollar estudios para aplicar el *software* antes mencionado en programas de capacitación de desarrollo profesional, especialmente de aquellos que trabajan en la atención primaria de salud.
